# The neural substrates of transdiagnostic cognitive-linguistic heterogeneity in primary progressive aphasia

**DOI:** 10.1186/s13195-023-01350-2

**Published:** 2023-12-16

**Authors:** Siddharth Ramanan, Ajay D. Halai, Lorna Garcia-Penton, Alistair G. Perry, Nikil Patel, Katie A. Peterson, Ruth U. Ingram, Ian Storey, Stefano F. Cappa, Eleonora Catricala, Karalyn Patterson, James B. Rowe, Peter Garrard, Matthew A. Lambon Ralph

**Affiliations:** 1 Medical Research Council Cognition and Brain Sciences Unit, University of Cambridge, 15 Chaucer Road, Cambridge, CB2 7EF UK; 2 Department of Clinical Neurosciences and Cambridge University Hospitals NHS Trust, University of Cambridge, Cambridge, UK; 3 Molecular and Clinical Sciences Research Institute, St. George’s, University of London, London, UK; 4 Division of Psychology and Mental Health, University of Manchester, Manchester, UK; 5 IUSS Cognitive Neuroscience Center (ICoN), University Institute of Advanced Studies IUSS, Pavia, Italy; 6 Dementia Research Center, IRCCS Mondino Foundation, Pavia, Italy

**Keywords:** Alzheimer’s disease, Frontotemporal dementia, Language, Network-based statistics, Voxel-based morphometry

## Abstract

**Background:**

Clinical variants of primary progressive aphasia (PPA) are diagnosed based on characteristic patterns of language deficits, supported by corresponding neural changes on brain imaging. However, there is (i) considerable phenotypic variability within and between each diagnostic category with partially overlapping profiles of language performance between variants and (ii) accompanying non-linguistic cognitive impairments that may be independent of aphasia magnitude and disease severity. The neurobiological basis of this cognitive-linguistic heterogeneity remains unclear. Understanding the relationship between these variables would improve PPA clinical/research characterisation and strengthen clinical trial and symptomatic treatment design. We address these knowledge gaps using a data-driven *transdiagnostic* approach to chart cognitive-linguistic differences and their associations with grey/white matter degeneration across multiple PPA variants.

**Methods:**

Forty-seven patients (13 semantic, 15 non-fluent, and 19 logopenic variant PPA) underwent assessment of general cognition, errors on language performance, and structural and diffusion magnetic resonance imaging to index whole-brain grey and white matter changes. Behavioural data were entered into varimax-rotated principal component analyses to derive orthogonal dimensions explaining the majority of cognitive variance. To uncover neural correlates of cognitive heterogeneity, derived components were used as covariates in neuroimaging analyses of grey matter (voxel-based morphometry) and white matter (network-based statistics of structural connectomes).

**Results:**

Four behavioural components emerged: general cognition, semantic memory, working memory, and motor speech/phonology. Performance patterns on the latter three principal components were in keeping with each variant’s characteristic profile, but with a spectrum rather than categorical distribution across the cohort. General cognitive changes were most marked in logopenic variant PPA. Regardless of clinical diagnosis, general cognitive impairment was associated with inferior/posterior parietal grey/white matter involvement, semantic memory deficits with bilateral anterior temporal grey/white matter changes, working memory impairment with temporoparietal and frontostriatal grey/white matter involvement, and motor speech/phonology deficits with inferior/middle frontal grey matter alterations.

**Conclusions:**

Cognitive-linguistic heterogeneity in PPA closely relates to individual-level variations on multiple behavioural dimensions and grey/white matter degeneration of regions within and beyond the language network. We further show that employment of transdiagnostic approaches may help to understand clinical symptom boundaries and reveal clinical and neural profiles that are shared across categorically defined variants of PPA.

**Supplementary Information:**

The online version contains supplementary material available at 10.1186/s13195-023-01350-2.

## Background

Primary progressive aphasias (PPA) are a heterogeneous group of neurodegenerative disorders of language [1, 2]. Three principal clinical variants are described: a semantic variant (svPPA or semantic dementia) displaying profound conceptual knowledge degradation and anterior temporal degeneration [3, 4], a nonfluent/agrammatic variant (nfvPPA) showing marked agrammatism and/or motor-speech difficulties with fronto-insular degeneration [5], and a logopenic variant (lvPPA) characterised by slowed spontaneous speech, phonological errors, and poor length-dependent sentence repetition, and left temporoparietal degeneration [6]. Within this taxonomy, associations between discrete symptoms and brain regions suggest relatively straightforward PPA characterisation; however, three emergent issues paint a more complex picture. First, PPAs show considerable clinical variation within, and overlap between, subtypes, with some features shared across distinct variants. Second, it is unclear why some patients present with additional, co-occurring non-linguistic cognitive impairments. Finally, we lack a full understanding of the neurobiological mechanisms underpinning cognitive-linguistic heterogeneity. Tackling these three issues is important to ensure diagnostic accuracy, identify potential behavioural/brain moderators of PPA disease phenotype, and to improve clinical trials design.

Language and semantic tests are central to the clinical characterisation of PPA. While marked and relatively selective conceptual knowledge degradation is most closely associated with svPPA [3, 7], approximately 40% of PPA cases show linguistic profiles falling between syndromic boundaries [7–9]. Particularly, disentangling nfvPPA from lvPPA on language performance alone can be challenging [10, 11]. Difficulties with word-finding, multisyllabic repetition, and lexical/phonological processing, typical of lvPPA, are also documented in nfvPPA [11–15]. Such overlaps emerge from partly dissociable neurocognitive substrates. For example, nfvPPA and lvPPA show compromised speech production but due to differential breakdowns in motor-speech/phonology/syntax vs. verbal working memory processing regions [11, 16–18]. Likewise, naming deficits may relate to disproportionate involvement of semantic (svPPA), phonological/motor-speech (nfvPPA), or phonological/working memory processing (lvPPA) regions [19]. These findings suggest that language profiles between syndromes vary in a graded, not absolute manner, closely reflecting involvement of different neurocognitive systems [7, 18, 20, 21]. Capturing such heterogeneity requires sensitive assessments capable of disentangling interdependencies at cognitive-neural process-levels to reveal shared/unique contributors. Currently, many measures used for PPA diagnostics derive metrics of overall aphasia severity and/or have limited range and depth of assessment [22–24]; therefore, they may poorly specify PPA type [10, 25] and breakdowns in corresponding neurocognitive systems [26]. As such, we require measures better suited to reveal process-level breakdowns common/unique to variants.

The second issue pertains to the status of non-linguistic cognition in PPA. Language and communication difficulties are central to lived experiences of PPA; unsurprisingly, these domains have received overwhelming research focus. Traditionally, non-linguistic difficulties were proposed to emerge either later with disease progression or as a by-product of primary aphasia [27]. Mounting evidence challenges this hypothesis to show general cognitive difficulties in early disease stages and at first clinic visit for many patients [28]. For example, transmodal semantic degradation in svPPA causes non-verbal semantic impairments even in early disease stages [29]. In nfvPPA, executive deficits often co-occur early with motor-speech difficulties [30]. LvPPA frequently displays non-linguistic cognitive difficulties such as nonverbal episodic memory, spatial orientation and working memory, and visuospatial processing [31–37]. In lvPPA, these deficits can emerge independent of disease severity, aphasia magnitude, and relate closely to encroachment of pathology into the temporoparietal cortex [20, 38, 39]. To understand PPA phenotypic heterogeneity, we need deeper investigation into non-linguistic dysfunction, its association with aphasia, and neurodegeneration profiles.

Finally, PPAs have been conceptualised as neural-network disorders where neurodegeneration spreads from syndrome-specific epicentres to functionally/structurally connected regions [40–44]. This account signals the need to evaluate concurrent changes to grey and white matter integrity to arrive at a comprehensive view of PPA clinico-anatomical changes. While grey matter correlates of PPA linguistic profiles are widely investigated [45], white matter changes and their relationship with linguistic/non-linguistic variation in PPA remain less understood. Previous work employing diffusion tensor imaging has revealed, in each variant, pronounced white matter changes to atrophy epicentres and their structurally connected regions [46–50]. Structural integrity between temporal, prefrontal, and parietal cortices further correlates with emergent language, behaviour, and episodic memory difficulties in PPA suggesting that clinical variability emerges from white matter damage beyond the language network [31, 51–55]. Diffusion tensor imaging, however, holds severe limitations in modelling crossing fibres (present in > 90% of white matter voxels) [56], thereby affecting false negative/positive results and interpretation of surrogate white matter integrity markers (e.g., fractional anisotropy, FA) [56, 57]. Addressing these limitations, we explored grey/white matter brain changes underlying PPA cognitive-linguistic heterogeneity, combining grey matter and contemporary white matter imaging analytic pipelines that reliably model intra-voxel directional white matter integrity.

Here, we make three advances towards an improved clinico-anatomical understanding of PPA phenotypic heterogeneity. First, we used multi-site data from PPA specialist clinics and a novel error-based assessment (Mini Linguistic State Examination; MLSE), designed for PPA, that holds proven sensitivity/specificity (> 95% accuracy) in characterising nuanced language profiles [58–60]. Compared to global performance scores, error patterns offer improved precision in revealing breakdowns in cognitive-linguistic processes and corresponding neural architectures [21, 61–63]. We also included an established general cognitive assessment (Addenbrooke’s Cognitive Examination-III, ACE-III) [64] showing demonstrable sensitivity to subtle non-linguistic cognitive changes in PPA [65]. Second, we modelled corresponding associations with grey/white matter integrity using whole-brain voxel-based morphometry (grey matter) and structural connectomic network-based statistics (white matter) derived from constrained spherical deconvolution-informed whole-brain tractography [57, 66–69]. The final advance is relating PPA phenotypic heterogeneity to breakdowns at the level of neurocognitive systems. Classic methods examining heterogeneity (e.g., group difference analyses) inadequately capture features cutting across diagnostic entities, within-group variability, and atypical/intermediate clinical presentations. Instead, multiple recent studies across a variety of neurological disorders have demonstrated the power of transdiagnostic multidimensional phenotypic geometries that (i) simultaneously model performance covariance patterns across groups/tests to uncover features shared between and specific to clinical entities and (ii) can help unpick process-level breakdowns contributing to overt test performance. This approach opens up a potentially powerful way to model clinical feature overlap by assimilating paradigmatic cases of each group, the graded variations within and between groups, and the many “mixed” cases presenting in the clinic, all within one multidimensional “geometry” [7, 20, 21, 70–75]. As these dimensions can reflect core neurocognitive systems, positioning individuals within this space further aids understanding of the blended mixture of damage to key neurocognitive systems that give rise to phenotypic similarity/differences [76]. In PPA, a number of studies have established this approach at the behavioural level [7, 20, 72], and so in this study, we take an important new step, by exploring how these multidimensional neurocognitive geometries map on to underlying neuroanatomy. To make progress towards translation and adoption of these frameworks into PPA clinical characterisation, we need to understand how emergent dimensions map on to common/different underlying brain systems. By combining a behaviour-brain dimensional mapping approach, we advance our current understanding of the genesis of PPA clinical heterogeneity and associated dysfunction of distributed neurocognitive systems.

## Methods

### Participants

We included 47 PPA patients (15 nfvPPA, 13 svPPA, and 19 lvPPA) diagnosed as per current criteria [1] based on comprehensive clinical review, neuropsychological examination, and structural magnetic resonance imaging (MRI). As a comparison group, 43 healthy control participants were recruited through the National Institute for Health Research “Join Dementia Research” register in Cambridge and London, patients’ relatives, and via local advertisement. Inclusion criteria for Controls comprised: aged between 40 and 75 years, absence of subjectively reported cognitive decline and/or a diagnosis of any pathological process causing a cognitive disorder, English as a first language, normal or corrected-to-normal hearing and vision, and willingness to participate in a study of language changes and dementia.

All participants provided written informed consent. Study ethics approval was obtained from the London-Chelsea Research Ethics Committee (REC#16/LO/1735). The Universities of London (St. George’s), Cambridge, and Manchester sponsored this study.

### Cognitive-linguistic assessment

All participants completed the MLSE (full details of the freely available test are in Patel et al. [58]). At the outset, we clarify that MLSE performance was not used to classify patients into their respective PPA categories; classification was done as per current diagnostic criteria and supportive features, as outlined in the previous section.

The MLSE quantifies the overall profile of language impairment based on the number and nature of errors made across 11 subtests. These subtests comprise (i) picture naming, (ii) repetition of syllables and multisyllables, (iii) word repetition and single word comprehension, (iv) non-word repetition, (v) non-verbal semantic association, (vi) verbal sentence comprehension, (vii) pictorial sentence comprehension, (viii) word and non-word reading, (ix) sentence repetition, (x) writing, and (xi) picture description. On each subtest, the MLSE quantifies accuracy as well as five types of potential errors (each reflecting an error typically made by one/more variant(s)): motor-speech, semantic, phonological, syntactic, and auditory-verbal working memory errors. Resultant scores from these error domains formed the main MLSE measures of interest and reflect performance across different facets of linguistic competence. An overall MLSE score, reflecting global language status, can also be derived. In the previous work, the MLSE has been shown to have ~ 96% predictive accuracy in discriminating PPA variants [58], lending confidence in its capacity to pick out subtle language changes in these variants. PPA patients also completed the ACE-III [64] including its subtests of attention, verbal memory, verbal fluency, language, and visuospatial functions. Subdomain scores from both tests formed behavioural measures of interest.

### Imaging

All participants underwent T_1_-weighted structural and 64-direction diffusion-weighted MRI (b-value = 1000 s/mm^2^). Full details of image acquisition, stratified by testing site, are in Supplementary Methods.

Whole-brain grey matter changes were indexed using voxel-based morphometry (VBM) analyses of structural T_1_-weighted MRI, integrated into Statistical Parametric Mapping software (SPM12: Wellcome Trust Centre for Neuroimaging, https://www.fil.ion.ucl.ac.uk/spm/software/spm12/). A standard pre-processing pipeline was implemented involving (i) brain segmentation into three tissue probability maps (grey matter, white matter, cerebrospinal fluid), (ii) normalisation (using diffeomorphic anatomical registration through exponentiated lie algebra, DARTEL) [77], (iii) study-specific template creation using grey matter tissue probability maps, (iv) spatial transformation to Montreal Neurological Institute (MNI) space using transformation parameters from the corresponding DARTEL template, and (v) image modulation and smoothing using 8-mm full-width-half-maximum Gaussian kernel to increase signal-to-noise ratio. Segmented, normalised, modulated, and smoothed grey matter images for all participants were concatenated into a four-dimensional grey matter image for VBM analyses.

Diffusion MRI data were preprocessed using a combination of MRtrix3 [78], FSL (https://fsl.fmrib.ox.ac.uk/fsl/fslwiki/), ANTs [79], and synthesized b0 for diffusion distortion correction (Synb0-DISCO) [80] packages. Standard preprocessing steps implemented within MRtrix3 (https://www.mrtrix.org/) and the BATMAN tutorial [81] were followed using commands built into MRtrix3 or interfacing with external software packages (e.g., FSL v6.0). Full description of pre-processing steps are detailed on the MRtrix3 webpage (https://mrtrix.readthedocs.io/en/latest/) and the BATMAN tutorial [81]. Briefly, they included (i) Marchenko-Pastur PCA denoising [82], (ii) Gibbs ringing artefact correction [83], (iii) field map estimation using Synb0-DISCO, (iv) motion, eddy, and b0 field corrections using eddy [84, 85], (v) B1 bias field correction using N4 correction [86], (vi) brain mask estimation, (vii) estimation of response functions for all tissue classes (grey matter, white matter, cerebrospinal fluid) [87], (viii) upsampling to 1.5-mm isotropic voxels and estimation of fibre orientation distribution (using single shell three-tissue constrained spherical deconvolution) [88], and (ix) multi-tissue informed log-domain intensity normalisation [89].

Whole-brain tractography was performed using MRtrix3 with anatomically constrained priors with back tracking (using *5ttgen* function on T1-weighted image) and the iFOD2 algorithm. We obtained 10 million streamlines per subject with a maximum streamline length of 250 mm. All other parameters were set to default. Seed points were determined dynamically using spherical-deconvolution informed filtering of tractograms (SIFT) model to improve distribution of reconstructed streamline density. Tractograms were further filtered using SIFT2 to improve quantification and biologically meaningful nature of whole-brain connectivity.

We constructed connectomes to quantify structural interconnectedness between brain regions and examine their associations with PPA task performance [90, 91]. Structural connectomes were constructed in MRtrix3 [78] using the Automated Anatomical Labelling-116 atlas [92] parcellation image that divides cortical, subcortical, and cerebellar regions into 116 nodes. Structural connectomes were constructed using the *tck2connectome* function with default parameters for measures of connectivity strength (streamline count or SC; total number of streamlines between two nodes) and integrity (FA; degree of overall anisotropy calculated as a weighted product of the FA-specific scalar image and SC) (both unthresholded to strongest weights) susceptible to changes early in the neurodegenerative process (see Supplementary Methods).

### Statistical analyses

Statistical analyses were conducted using a combination of RStudio v4.0.3 [93], MATLAB-R2018 [94], and MRtrix3 v3.0.4 [78].

#### Behavioural data analyses

##### Demographic and neuropsychological variables

Binomially distributed variables were analysed using chi-squared tests. For continuous variables, we examined normality of distribution using Shapiro–Wilk tests and box-and-whisker plots, followed by analysis of variance (ANOVA) with Sidak corrections for post hoc comparisons for small sample sizes. Alpha levels were set at *p* ≤ 0.05 for overall group comparisons and at *p* ≤ 0.025 for post-hoc comparisons. Effect sizes were reported using partial eta-squared values (${\eta }_{p}^{2}$) with 95% confidence intervals. For brevity, we report test statistics for all comparisons in Table 1.

**Table 1:** Demographic, clinical and neuropsychological assessment performance for all groups

	svPPA	nfvPPA	lvPPA	Control	Magnitude of group effect	Direction of post-hoc effect
*N*	13	15	19	43	-	-
Recruitment site (C/M/S)	5/1/7	9/2/4	5/2/12	23/0/20	-	-
Sex (M: F)	7:6	10:5	6:13	22:21	$\chi$ ^2^(3) = 4.3; *p* = .22	-
Age (years)	66.3 (5.4)	71.2 (5.6)	70.6 (7.7)	61(9.4)	*F*(3,86) = 9.4; ***p* < .001**; ${\eta }_{p}^{2}$=.25[.1–1]	Control < lvPPA, nfvPPA
Education (years)	18.9 (2.3)	17.2 (1.9)	19.2 (3)	21.1 (3)	*F*(3,86) = 7.9; ***p* < .001**; ${\eta }_{p}^{2}$=.22[.09–1]	nfvPPA < Controls
Handedness (R: L)	13:0	13:2	18:1	39:4	$\chi$ ^2^(3) = 2; *p* = .56	-
Symptom duration (years)	1.6 (1.9)	.7 (.7)	3.3 (5.5)	-	*F*(2,44) = 2.5; *p* = .09; ${\eta }_{p}^{2}$=1[0–1]	-
Cognitive and language assessments						
MLSE total score (100)	73.4 (12.6)	64.2 (24.4)	79.5 (5.1)	98.6 (1.7)	*F*(3,86) = 42.7; ***p* < .001**; ${\eta }_{p}^{2}$=.6[.49–1]	Patients < Controls
ACE-III Total (100)	50.8 (19.1)	55 (24.5)	54.8 (8.8)	-	*F*(2,35) = .22; *p* = .8; ${\eta }_{p}^{2}$=.01[0–1]	-
ACE-III Attention total (18)	10.7 (4.5)	11.8 (5.2)	14.6(2.1)	-	*F*(2,35) = 2.7; *p* = .07; ${\eta }_{p}^{2}$=.014[0–1]	-
ACE-III Memory total (26)	7.8 (6.5)	11.9 (8.2)	9.2 (4.5)	-	*F*(2,35) = 1.2; *p* = .2; ${\eta }_{p}^{2}$=.07[0–1]	-
ACE-III Fluency total (14)	3.3 (2.5)	3.4 (3.2)	4.4 (1.8)	-	*F*(2,35) = .6; *p* = .53; ${\eta }_{p}^{2}$=.04[0–1]	-
ACE-III Language total (26)	17.5 (6.2)	16.2 (7)	11.6 (3.2)	-	*F*(2,35) = 3.4; ***p* = .042;** ${\eta }_{p}^{2}$=.16[0–1]	svPPA < lvPPA
ACE-III Visuospatial total (16)	11.3 (3)	11.5 (5.2)	14.8 (1.4)	-	*F*(2,35) = 3.5; ***p* = .039;** ${\eta }_{p}^{2}$=.17[0–1]	lvPPA < svPPA

Maximum test scores reported in brackets. For recruitment site, *C* Cambridge, *M* Manchester, *S* St. George’s, University of London. For all groups, mean and standard deviations reported; $\chi$ ^2^ = Chi-square value; for magnitude of group effect, exact $\chi$ ^2^/*F-*statistics, exact *p*-values (unless *p* < .001), and effect size (${\eta }_{p}^{2}$) values reported; for group effect statistical comparisons, *p*-values are in bold if *p* ≤ .05; post hoc alpha threshold set at *p* ≤ .025

*ACE-III* Addenbrooke’s Cognitive Examination-III, *MLSE* Mini Linguistic State Examination, *lvPPA* logopenic variant primary progressive aphasia, *nfvPPA* nonfluent variant primary progressive aphasia, *svPPA* semantic variant primary progressive aphasia

##### Data handling and PCA

We used principal component analysis (PCA) that enables modelling of shared cognitive computations/dimensions underlying task performance. First, missing data were tabulated (ACE-III scores missing for 4 nfvPPA, 3 lvPPA, 2 svPPA) and imputed using fourfold cross-validated probabilistic PCA (see Supplementary Methods and reference [95]). This method robustly handles ~ 25% missing data and guards against overfitting [96, 97]. On this “full” dataset, effects of testing site were regressed out using linear models and residuals were carried forward for subsequent analyses. To guide extraction of optimal number of PCA components, we used four-fold cross-validated component selection methods (10 iterations) [95] with Venetian blind sample shuffling at each iteration to control for participant order effects (Supplementary Methods). The solution with the average lowest root-mean-squared-error value across iterations, along with standard scree plot criteria (eigenvalues ≥  ~ 1.0) [98], guided the number of components to be extracted.

The PCA model was considered adequate (Kaiser–Meyer–Olkin statistic = 0.65). Behavioural data from the PPA group (*N* = 47) were entered into a varimax-rotated PCA. Orthogonal rotations maximise dispersion of loadings, allow little variance to be shared between components, and promote a simple structure and clear behavioural interpretation of results. Components were given labels reflecting the majority of tests loading heavily (loadings >|.5|). Labels function as short hands referring to high loading tests on components; they facilitate ease of reporting, although they by no means reflect the entirety of cognitive computations captured within components. Component scores were subject to group comparisons and correlations with symptom duration (two-tailed Pearson’s *r*) and brain changes.

#### Grey matter analyses

Between participant groups, voxel-wise changes in grey matter intensity were assessed using independent *t*-tests, with age, total intracranial volume, and testing site included as nuisance variables. Sex did not form an a priori variable of interest in neuroimaging analyses, therefore, we did not include it as a nuisance variable. In subsequent follow-up analyses, we further found no significant effects of sex on emergent PCs (Component 1: *t* =  − 0.2, *p* = 0.8; Component 2: *t* = 0.8, *p* = 0.3; Component 3: *t* =  − 0.2, *p* = 0.8; Component 4: *t* =  − 0.1, *p* = 0.8), therefore, did not include it as a nuisance variable in neuroimaging correlation analyses. Clusters were extracted using the threshold-free-cluster-enhancement (TFCE) method [99] corrected for family-wise error (FWE) at *p* < 0.05 with a cluster threshold of 100 contiguous voxels.

In the PPA group (*N* = 47), we performed VBM correlation analyses to examine associations between PCA component scores and whole-brain changes in grey matter intensity. A correlation-only statistical model using *t*-contrasts was implemented. Age, total intracranial volume, and testing site were included as nuisance variables. Anatomical locations of statistical significance were overlaid on the MNI standard brain and maximum co-ordinates in MNI space were indexed. Clusters were extracted using a threshold of *p* < 0.001 uncorrected for multiple comparisons with a cluster threshold of 50 contiguous voxels to capture changes in smaller subcortical structures.

#### White matter analyses

We performed network-based statistics (NBS) [100] (implemented within MRTrix3) to identify subnetworks (connection clusters comprising sets of interconnected edges) where SC/FA connectivity statistically differ between groups. Briefly, NBS computes independent *t*-test statistics at each edge (i.e., connections between two nodes) followed by statistical thresholding to identify subnetworks (clusters of connected edges) varying between groups/associated with a covariate of interest [100]. Critical *t*-values were set at 4, significant subnetworks were identified using the TFCE method [101] and corrected for FWE at *p* < 0.05. Age and testing site were included as nuisance covariates. Sex did not form an a priori variable of interest in our analyses, therefore, we did not include it as a nuisance variable in any neuroimaging analyses.

In the PPA group (*N* = 47), NBS correlation analyses (*t*-contrasts) were performed to examine associations between PCA component scores and whole-brain changes in white matter integrity (for SC and FA, separately) with age and testing site included as nuisance variables. Subnetworks of statistical significance were extracted using a threshold of *p* < 0.001 uncorrected for multiple comparisons, overlaid on the MNI standard brain with maximum co-ordinates in MNI space, and visualised using the xjView toolbox (www.alivelearn.net/xjview/) and BrainNetViewer [102].

### Data availability

Ethical requirements to ensure patient confidentiality precludes public archiving of our data but non-identifiable derived data can be provided on request to bona fide researchers. A data transfer agreement may be required if potentially identifiable data are requested, including raw clinical and structural imaging data. Researchers who would like to access data should contact the senior author (M.A.LR).

## Results

### Behavioural analyses

#### Demographic, clinical and cognitive performance

Between participant groups, no significant differences emerged for sex or handedness distribution (*p* > 0.10) or symptom duration (*p* = 0.09) (Table 1). Relative to Controls, nfvPPA and lvPPA groups were significantly older and the nfvPPA group had significantly fewer years of education (all *p* < 0.001). On overall language performance (MLSE Total score), patient groups performed significantly more poorly than Controls (all *p* < 0.001) but comparably to each other (*p* > 0.1). On MLSE subdomains, findings largely concurred with each variant’s descriptive template (i.e., disproportionately greater semantic errors in svPPA, motor-speech errors in nfvPPA, auditory-verbal working memory errors in lvPPA) (Supplementary Results). Patient groups further displayed comparable performance on the ACE-III total and its subdomains of attention, memory, and fluency. Significant differences emerged on the ACE-III Language total, where svPPA patients displayed significantly poorer performance than the lvPPA group (*p* = 0.016). The inverse pattern was noted on ACE-III Visuospatial total, with lvPPA patients exhibiting disproportionately poorer performance relative to svPPA (*p* = 0.001).

#### Determining principal components underlying cognitive heterogeneity

**Fig. 1:**
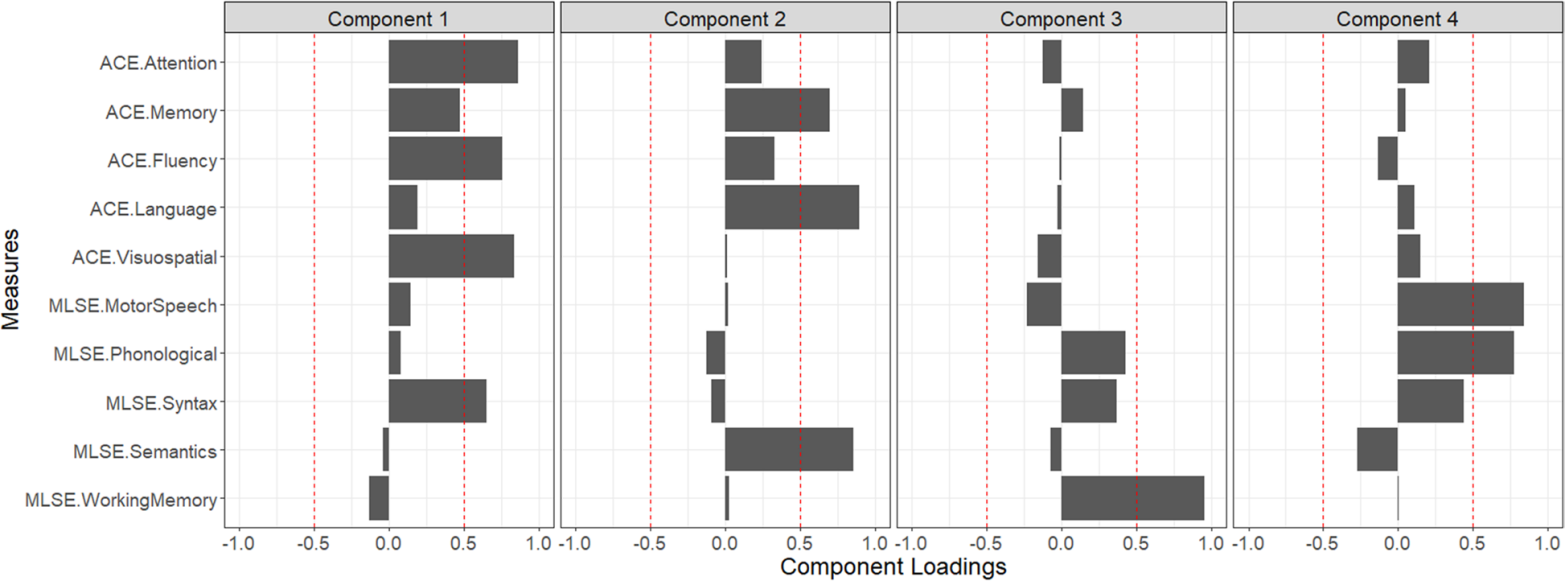
Component loadings for cognitive measures from varimax PCA in the combined PPA group (*N* = 47). Figure indicates emergent cognitive factors, with components ordered by amount of overall variance explained. Red dashed lines represent component loading cut-offs (|0.5|). ACE-III Addenbrooke’s Cognitive Examination-III, MLSE Mini Linguistic State Examination, PCA principal component analysis, PPA primary progressive aphasia

Component selection and scree plot analyses converged on a four-component solution (Fig. 1) (eigenvalues > 0.9) explaining 82.2% of performance variation (Supplementary Figs. 1–3). Component 1 explained 36.2% of the overall variance and loaded positively on ACE-III Attention, Fluency, Visuospatial subscales and MLSE syntax scores. This component was referred to as ‘general cognition’. Component 2 was labelled ‘semantic memory’, captured 22.7% of overall variance and loaded positively on the ACE-III Memory, Language subscales and the MLSE Semantics subdomain. Component 3 was titled ‘working memory’, loaded positively on the MLSE verbal Working Memory subscale and accounted for 14.1% of overall performance variance. Finally, component 4 was named ‘motor speech/phonology’ as it loaded positively on the MLSE Motor Speech and Phonological subscales and explained 9.3% of overall performance variance.

#### Graded overlaps and differences in PPA cognitive-linguistic performance

**Fig. 2:**
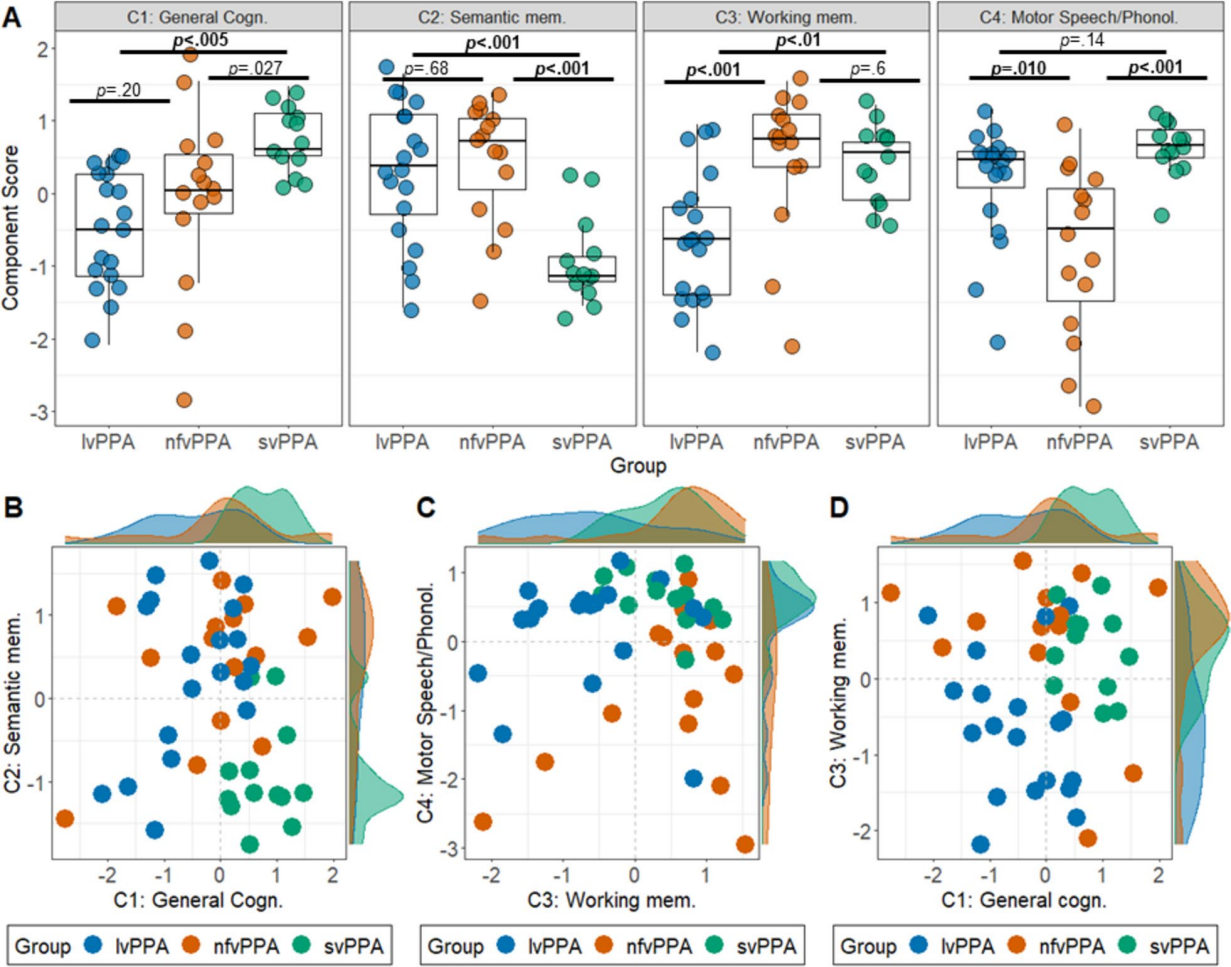
PPA performance on emergent components.** A** Group differences on emergent components from the varimax PCA. Statistical comparisons run using ANOVA with post hoc comparisons using Sidak corrections (alpha cut-off at *p* = .025; all relevant statistics displayed in the ‘ Results’ section corresponding to this figure). Bolded *p*-values indicate statistically significant differences. Scatter plots with marginal density histograms for select components displaying relationships between **B** general cognition (Component 1) and semantic memory (Component 2), **C** working memory (Component 3) and motor speech/phonology (Component 4), and **D** general cognition (Component 1) and working memory (Component 3). Positive scores indicate better performance. C component from PCA, PCA principal component analysis, lvPPA logopenic variant primary progressive aphasia, nfvPPA nonfluent variant primary progressive aphasia, svPPA semantic variant primary progressive aphasia

Significant group differences were noted on the general cognition component [*F*(2,44) = 7.4; *p* = 0.001; ${\eta }_{p}^{2}$= 0.25[0.07–1]] where the lvPPA group performed significantly worse than the svPPA group (*p* = 0.002), while nfvPPA patients displayed intermediate performance with considerable inter-individual variation (Fig. 2A-D). On the semantic memory component, significant group differences were found [*F*(2,44) = 11.3; *p* < 0.001; ${\eta }_{p}^{2}$= 0.34[0.15–1]] with poorest performance in svPPA relative to nfvPPA/lvPPA (all *p* < 0.001). On the working memory component, significant group differences were noted [*F*(2,44) = 8.9; *p* < 0.001; ${\eta }_{p}^{2}$= 0.29[0.1–1]], with the lvPPA group performing significantly more poorly in comparison to svPPA/nfvPPA (all *p* < 0.01). Scatter plots indicated that the majority of lvPPA patients visually separated from nfvPPA/svPPA when combining working memory and general cognition performance (Fig. 2D). Finally, significant group differences emerged on the motor speech/phonology component [*F*(2,44) = 10; *p* < 0.001; ${\eta }_{p}^{2}$= 0.31 [0.12–1]] where nfvPPA displayed disproportionately greater deficits (all *p* ≤ 0.01) with comparable performance between lvPPA and svPPA.

#### Associations between PCA components and disease duration

In the whole PPA group, no significant associations emerged between disease duration and PCA components (all *r* < 0.11 and >  − 0.24; all *p* > 0.10) (Supplementary Table 1).

### Neuroimaging analyses

#### Group differences in grey matter intensity (VBM)

Relative to Controls, svPPA patients showed significantly reduced grey matter intensity in bilateral anterior temporal lobes (left > right), inferior/middle/superior temporal gyri, and medial temporal lobes. Compared to Controls, nfvPPA displayed significantly reduced grey matter intensity in bilateral inferior/middle frontal gyrus, anterior cingulate and insula (all left > right) extending into left inferior/middle/superior temporal gyri and medial temporal cortices. In the lvPPA group, relative to Controls, significant reductions in grey matter intensity were noted in the left temporoparietal junction/inferior parietal lobule, inferior/middle/superior temporal gyri, extending to the left posterior parieto-occipital and cerebellar cortices, right temporoparietal cortices, and bilateral frontal and medial temporal regions located in close proximity to the Sylvian fissure. Between-patient comparisons revealed no significant clusters (Supplementary Table 2 and Supplementary Fig. 4).

#### Group differences in white matter integrity (NBS)

Detailed descriptions of NBS findings are in Supplementary Results and Supplementary Figs. 5–6. Considering SC first, relative to Controls, svPPA displayed significantly reduced SC in temporal regions and connections to frontoparietal cortices (left > right). In nfvPPA, marked SC reductions in frontoinsular regions, intra-prefrontal, and frontal to temporoparietal connections were noted. In lvPPA, significant SC reductions were found in left temporoparietal/inferior parietal regions and their connections with fronto-temporal, parietal, occipital, and cerebellar nodes. Relative to nfvPPA/lvPPA, svPPA displayed greatest SC reduction in the left anterior temporal lobe. Comparisons between nfvPPA and lvPPA revealed significantly fewer streamlines between left frontoinsular regions in nfvPPA and SC reductions between fronto-cerebellar regions in lvPPA. For all patient-Control contrasts, findings for FA concurred with SC and extended to include a wider network of disconnections between bilateral anterior and posterior brain regions (see Supplementary Results).

#### Grey and white matter correlates of principal cognitive factors

We collate and report VBM and NBS correlation findings from the overall PPA group in a component-specific manner for better readability (see also Supplementary Table 3).

##### General cognition

**Fig. 3:**
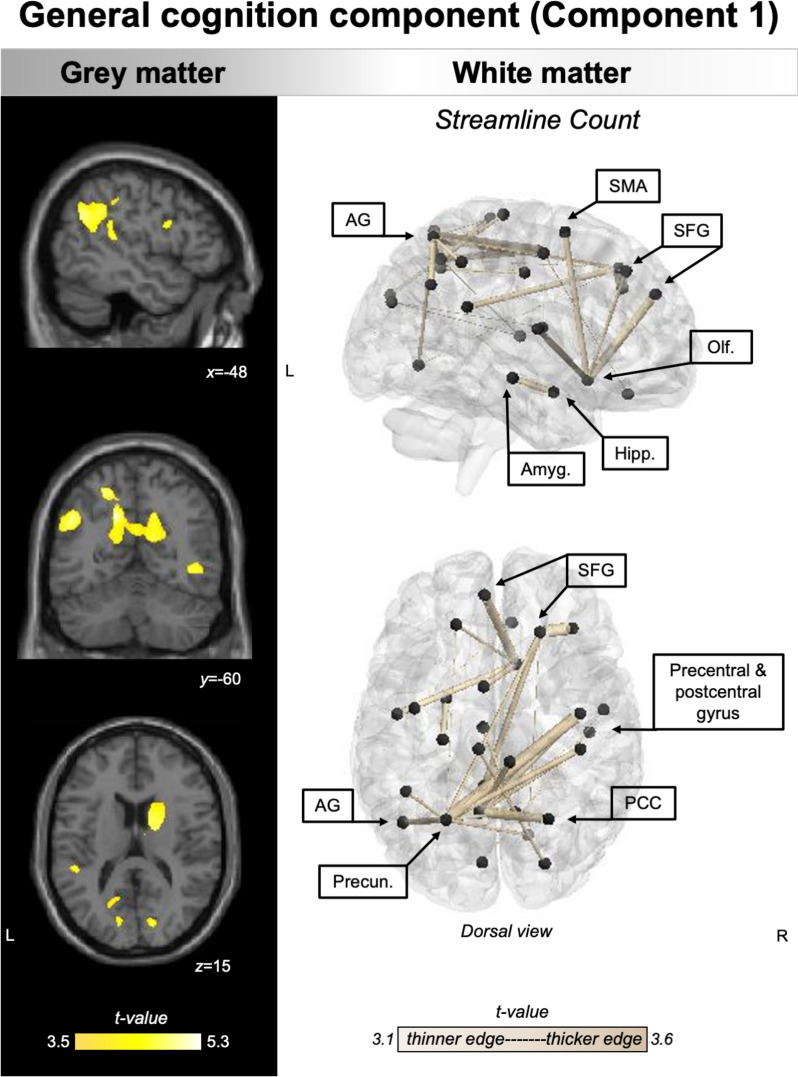
Regions of grey matter (left panel) and white matter (right panel) changes that correlate with general cognition component. PCA, grey, and white matter analyses were conducted in the combined PPA group (*N* = 47). For VBM, coloured voxels indicate regions that emerged as significant in the voxel-based morphometry analyses at a threshold of *p* < .001 uncorrected for multiple comparisons with a cluster threshold of 50 contiguous voxels. Age, total intracranial volume, and testing site were included as covariates in the analyses. Clusters are overlaid on the MNI standard brain with *x*, *y*, and *z* co-ordinates reported in MNI standard space. For NBS, black spheres indicate cortical nodes whose edges (gold lines) emerged significant at *p* < .001 uncorrected for multiple comparisons. Edge thickness corresponds to corresponding effect size (i.e., *t*-value). Age and testing site were included as covariates in the analyses. L left, R right, PCA principal component analysis, PPA primary progressive aphasia, AG angular gyrus, SMA supplementary motor area, SFG superior frontal gyrus, Olf. olfactory cortex, PCC posterior cingulate cortex, VBM voxel-based morphometry, NBS network based statistics

VBM and NBS converged to indicate general cognitive performance as relating to grey matter intensity changes in the left temporoparietal/inferior parietal regions, bilateral medial parieto-occipital, inferior frontal and subcortical regions, and SC changes between left temporoparietal/medial parietal regions, parieto-frontal connectivity, and intra-medial temporal connections (Fig. 3).

##### Semantic memory

**Fig. 4:**
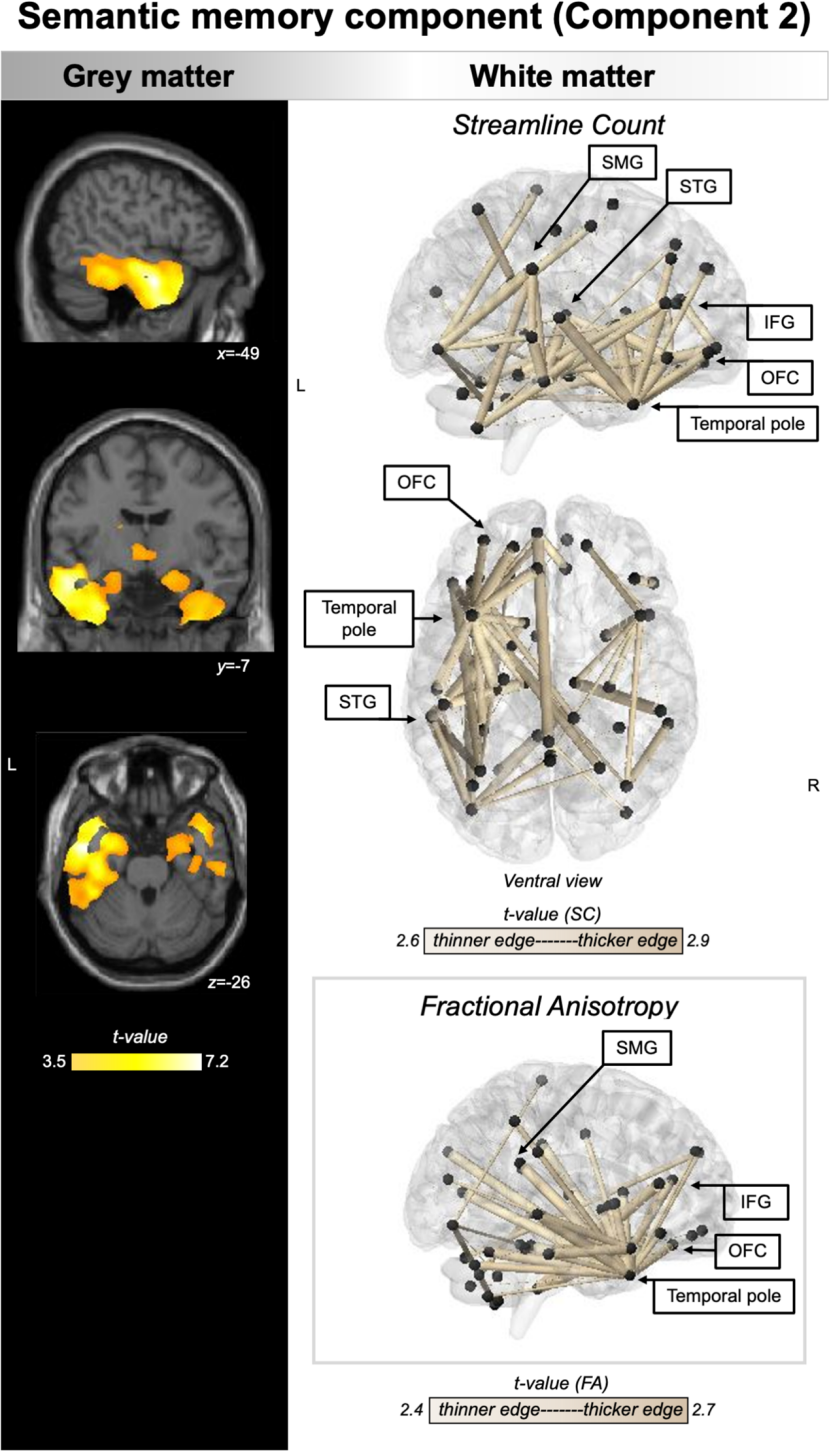
Regions of grey matter (left panel) and white matter (right panel) changes that correlate with semantic memory component. PCA, grey, and white matter analyses were conducted in the combined PPA group (*N* = 47). For VBM, coloured voxels indicate regions that emerged significant in the voxel-based morphometry analyses at a threshold of *p* < .001 uncorrected for multiple comparisons with a cluster threshold of 50 contiguous voxels. Age, total intracranial volume, and testing site were included as covariates in the analyses. Clusters are overlaid on the MNI standard brain with *x*, *y*, and *z* co-ordinates reported in MNI standard space. For NBS, black spheres indicate cortical nodes whose edges (gold lines) emerged as significant at *p* < .001 uncorrected for multiple comparisons. Edge thickness corresponds to corresponding effect size (i.e., *t*-value). Age and testing site were included as covariates in the analyses. L left, R right, PCA principal component analysis, PPA primary progressive aphasia, SMG supramarginal gyrus, STG superior temporal gyrus, IFG inferior frontal gyrus, OFC orbitofrontal cortex, VBM voxel-based morphometry, NBS network based statistics

VBM and NBS converged to indicate semantic memory performance as associated with grey matter and SC changes in bilateral anterior temporal (greater on the left), medial temporal (hippocampus, amygdala, and subcortical regions), and frontal regions (medial/inferior/orbito-frontal cortices) (Fig. 4). In addition, left-lateralised connectivity changes (SC and FA) within temporal cortex, temporoparietal, frontotemporal, and temporoparietal-cerebellar cortices emerged as associated with semantic memory performance.

##### Working memory

**Fig. 5:**
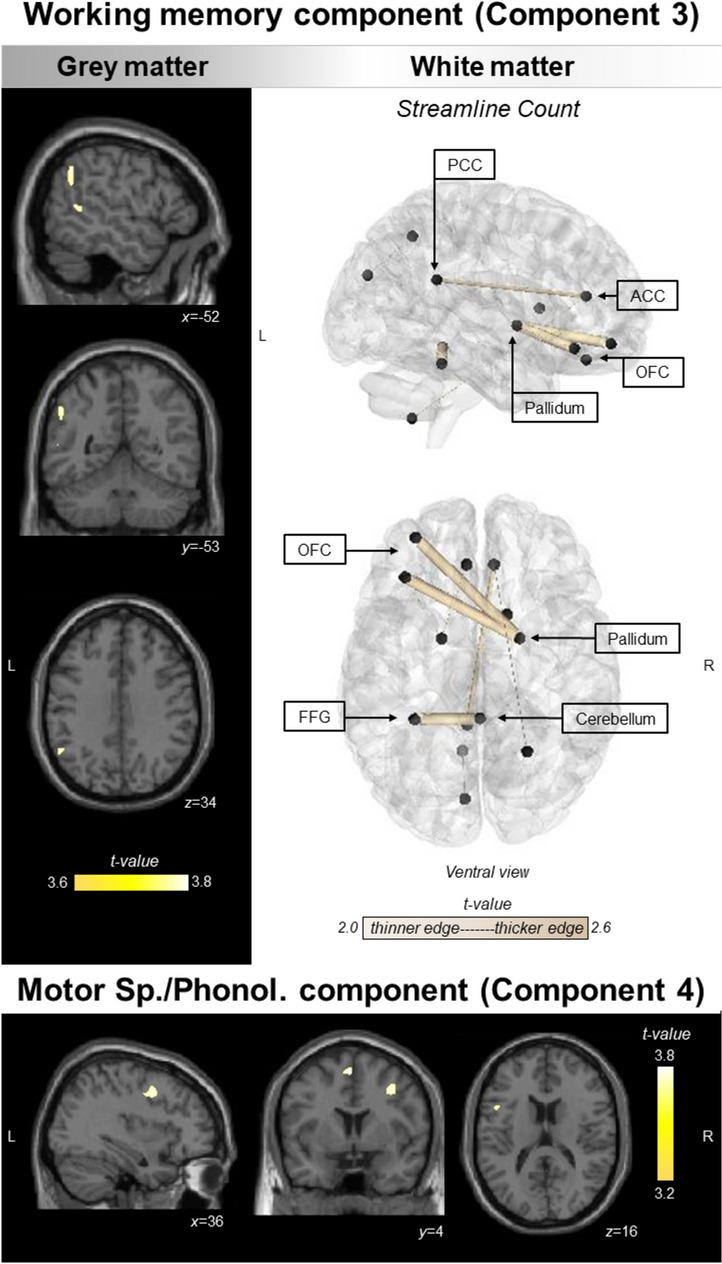
Regions of grey matter (top left panel and bottom panel) and white matter (top right panel) changes that correlate with working memory and motor speech/phonology components. Findings for working memory component displayed in top left and right panels and findings for motor speech/phonology component displayed in bottom panel. PCA, grey, and white matter analyses were conducted in the combined PPA group (*N* = 47). For VBM, coloured voxels indicate regions that emerged as significant in the voxel-based morphometry analyses at a threshold of *p* < .001 uncorrected for multiple comparisons with a cluster threshold of 50 contiguous voxels. Age, total intracranial volume, and testing site were included as covariates in the analyses. Clusters are overlaid on the MNI standard brain with *x*, *y*, and *z* coordinates reported in MNI standard space. For NBS, black spheres indicate cortical nodes whose edges (gold lines) emerged significant at *p* < .001 uncorrected for multiple comparisons. Edge thickness corresponds to corresponding effect size (i.e., *t*-value). Age and testing site were included as covariates in the analyses. No correlations emerged in the NBS for the Motor Speech/Phonology component. L left, R right, PCA principal component analysis, PPA primary progressive aphasia, PCC posterior cingulate cortex, ACC anterior cingulate cortex, OFC orbitofrontal cortex, FFG fusiform gyrus, VBM voxel-based morphometry, NBS network-based statistics

Working memory performance correlated with grey matter intensity changes in the left inferior parietal (supramarginal and angular gyrus) and posterior temporal cortices, and SC changes between cortical midline regions, and fronto-pallidal circuitry (Fig. 5).

##### Motor speech/phonology

Motor speech/phonology performance was associated with grey matter integrity of bilateral superior/middle/inferior frontal regions and precentral gyri (Fig. 5). No associations emerged with structural connectivity indices.

## Discussion

Understanding the neurocognitive drivers of PPA phenotypic heterogeneity will improve the diagnosis, identification of potential moderators of disease phenotype, and stratification of patients for trials. Consistent with recent explorations [21, 71, 72, 103], we showed that PPA phenotypic profiles vary along four orthogonal dimensions: general cognition, semantic memory, working memory, and motor speech/phonology changes. Each component was characterised by graded performance variations, including prototypical variant-specific deficits, but also overlap between categorically distinct syndromes. Performance on all components further emerged independent of disease duration. For the first time, we were able to explore and show that each phenotypical dimension was associated with distinct grey and white matter neural network changes in PPA. Although each component differed in its loading onto the three classically defined diagnostic groups, none were unique to a given syndrome.

Before discussing our results, it is important to contextualise the transdiagnostic approach. Current taxonomies classify PPA on mutually exclusive language profiles [1]. These rigid categorical boundaries leave little room to accommodate phenotypic variation; therefore, an ‘atypical’ case could be thought to signal a novel subtype or a mixed phenotype of a variant. For example, lvPPA cases showing cognitive-linguistic symptoms outside of their diagnostic template are proposed to represent a distinct subtype of the condition [104, 105]. Syndromic sub-classification is a reasonable pursuit provided there is sufficient within-group homogeneity and between-group differentiation that are consistently identifiable between studies and centres. However, increasing evidence points to systematic cognitive-linguistic differences of features within variants and overlap of features between variants, not consistently replicable across studies, hinting at fuzzy between-group boundaries and substantial within-group heterogeneity [21, 72, 74, 106]. Such inter-patient graded variations do not seem to arise from measurement noise or inaccurate differential diagnosis. Instead, these variations are systematic in nature and can be captured by transdiagnostic approaches, where constructing multidimensional geometries allows (a) prototypical, atypical, and mixed PPA cases to be located and related to each other and (b) as shown for the first time in this study, for these systematic dimensions to be related to the underpinning neurobiology in the form of grey matter atrophy and changes in structural connectivity in PPA.

On the first component, general cognition, lvPPA patients displayed disproportionate deficits relative to the other groups; we further noted marked inter-individual variation in nfvPPA performance. While studies deciphering the lvPPA phenotype have largely focused on its language profile, evolving clinical conceptualisations of the syndrome highlight a “multidimensional” cognitive profile, encompassing core language deficits with variable non-linguistic difficulties, emerging independent of disease severity and aphasia magnitude [38]. In fact, even in the earliest reported cases of lvPPA, non-linguistic changes, such as calculation difficulties, were noted [27]. By recent estimates, > 90% of lvPPA patients present with some magnitude of non-linguistic cognitive impairment [39] notable on calculation, praxis, episodic, and working memory [37, 38, 107]. This pattern of impairment, however, emerges in the context of marked within-group heterogeneity in the syndrome. For example, some studies show that two lvPPA patients, with similar levels of core language impairment can markedly diverge in their visuospatial, executive functions, and memory performance [20, 39]. Such patterns of systematic variation on non-linguistic cognitive performance in lvPPA emerge irrespective of severity of core language impairment [20]. A similar pattern was noted in the current study when contrasting general cognition with working memory profiles; irrespective of the magnitude of working memory deficits in lvPPA, marked variation in general cognitive performance was noted. Amidst relatively comparable symptom duration and MLSE total scores between PPA groups, our findings suggest systematic variation in general cognition performance in lvPPA that may be independent of disease duration or severity of language performance. To explain this systematic variation, new clinico-anatomical models point to temporoparietal cortices as shared neural substrates of cognitive-linguistic dysfunction in lvPPA [38], owing to the myriad domain-general/selective cognitive computations supported by these regions [108–112]. It is suggested that systematic variation on both core language and co-occurring variable non-linguistic cognitive deficits in the syndrome emerges from the stochastic spreading of pathology, functional aberrations, and atrophy from the temporoparietal epicentre in the syndrome [38]. Accordingly, lvPPA patients with greater parietal dysfunction, as opposed to those with temporal-dominant degeneration, show increased general cognitive, visuospatial, executive disturbances, and loss of functional autonomy [113, 114]. We extend this body of evidence to show that general cognitive changes in PPA relate to involvement of both grey and white matter networks centred on temporoparietal junction, inferior, and medial parietal cortices. While the implicated networks largely mirror lvPPA atrophy epicentres, encroachment of atrophy into posterior parietal regions magnifies cognitive/linguistic deficits in nfvPPA [26, 115], potentially explaining nfvPPA inter-individual performance variation noted here. It is further possible that performance in lvPPA and nfvPPA groups on the general cognition component was influenced by the additional loading of the MLSE Syntax subdomain. As syntactic comprehension has been reported as a core deficit in these PPA variants [116], it will be important for future work in PPA to untangle relationships between core linguistic syntax changes with co-occurring general cognitive changes.

Three additional components emerged in our analysis, each reflecting language changes prototypical of one/more PPA variant(s). Performance on the semantic memory component was poorest in svPPA and linked to grey/white matter degeneration of bilateral anterior/posterior temporal lobes. Multimodal evidence converges to spotlight the anterior temporal lobes as semantic processing hubs within a distributed frontal and temporo-parietal semantic network [117]. Degeneration of the semantic hub in the anterior temporal lobe causes profound conceptual degradation and greatly undermines functioning of the entire semantic network [42, 117–121]. Visually inspecting the distribution of scores on the semantic memory component, it is also noteworthy that a number of lvPPA patients also performed poorly on the semantic memory component. It is noteworthy that performance on semantic tasks can be failed not only because of a degradation of underlying representations (like in svPPA) but also because of failures in ancillary cognitive processes, an important one being “semantic control” [122]. Semantic control processes support access and manipulation of semantic information based on task and context demands and, at a neural level, localise to inferior frontal and temporoparietal regional integrity [117]. An emerging hypothesis suggests that lvPPA patients may fail semantic tasks due to deregulated semantic control (emerging from temporoparietal dysfunction) [38]. In support of this hypothesis, a case study of an lvPPA patient reported their difficulty with using and manipulating electrical appliances, despite intact conceptual knowledge for the items and their typical usage [123]. Although temporoparietal regions emerged in our study to correlate with performance on the semantic memory component, it is important for future work to probe the specific links between semantic control and temporoparietal integrity in lvPPA towards understanding the neurocognitive origins of semantic cognition difficulties in this syndrome. Another possibility in lvPPA is that a strong anomic profile in the syndrome may have led to a drop in performance on this component, especially as measures such as the ACE-III Language task loaded heavily on the semantic memory component. Turning to working memory, performance on this component was poorest in lvPPA and associated with left inferior parietal dysfunction and disconnections between cortical midline and prefrontal-basal ganglia circuits. Auditory-verbal working memory difficulties, a hallmark of lvPPA, are typically discussed in the context of temporoparietal involvement in the syndrome [6, 124]; however, these functions are supported by a network of prefrontal, cortical midline, striatal/basal ganglia, and temporoparietal regions that interact to gate, represent, retrieve, and update information within working memory [125–127]. Specifically, orbitofrontal and striatal dysfunction, notable in nfvPPA/svPPA, could contribute to verbal and nonverbal working memory difficulties noted in the syndrome [128], over-and-above primary motor-speech and semantic difficulties, respectively. Interestingly, the scatterplots suggested that a combination of working memory and general cognition components delineate the majority of lvPPA cases from svPPA/nfvPPA. Both components are putatively thought to stress temporoparietal cortex functions that are affected early in lvPPA [6, 38, 124]; their combination, therefore, could hold enhanced clinical utility in differentiating lvPPA. Finally, motor speech/phonology performance was greatly affected in nfvPPA and correlated with bilateral superior/inferior frontal regions underpinning these functions [5, 129]. The clustering of these error types suggests their co-occurrence in a number of nfvPPA cases [12, 14, 130, 131] where speech distortions may reflect phonetic errors due to apraxia of speech, while phonemic errors may reflect motor speech impairment/difficulties in phonemic selection in nfvPPA [132, 133]. In contrast, motor speech/phonology was relatively preserved in the svPPA group—a finding reported previously [7] and reflecting the neurocognitive divergence of phonological and semantic-processing regions in the brain [134].

Our study has several limitations. Capturing associations between biomarker and cognitive changes in PPA are important to inform targeted treatment and management efforts. Our sample size was relatively small and the majority of our sample did not have supportive biomarker evidence and/or have not yet come to autopsy, preventing clinico-pathological correlations. We also could not include a metric of disease severity. On this note, our findings hint at the importance of including a multidimensional metric of disease severity that captures the differing types, nature, and magnitude of language and general cognitive deficits in PPA. Such a measure may be more informative of multidimensional phenotypic changes with time in PPA, over a singular, unidimensional metric of disease severity. The prospective nature of our study also meant our PCA was constrained by the a priori planned behavioural assessments. Furthermore, white matter hyperintensities are markedly prevalent in older populations. In individuals with neurodegenerative disorders, moreover, functional brain changes precede structural alterations. Our study, however, did not have T_2_-weighted structural neuroimaging or task-based functional neuroimaging data available, precluding us from investigating contributions of vascular and functional brain changes. By including patients who fell within current categorical diagnostic boundaries of PPA, we show the presence of graded variation within and between syndromes. However, there is a future need to extend such work to mixed PPA cases, allowing to explore where such mixed presentations sit within this multidimensional space of structured phenotypic variation. The current study was limited in its capacity to address this issue due to the lack of qualitative/quantitative data on the specific clinical features that overlapped between our patient groups and the absence of a mixed PPA cohort. Although we found that none of our emergent components were unique to a given syndrome, the inclusion of mixed PPA patients will be important to grade the overlap of clinical features and better characterise the continuous dimension of performance variation between classical and intermediate PPA syndrome categories. Future transdiagnostic studies may benefit from inclusion of larger samples of both PPA (including mixed PPA) and non-PPA syndromes associated with neurodegeneration, including other phenotypic expressions of Alzheimer’s disease and movement disorders associated with aphasia [70], followed up over time from their first clinical examination. It will also be important for future work to include a broader neuropsychological battery to arrive at a more comprehensive understanding of performance variations along multiple dimensions of cognitive-behavioural change. This will be important to understand common mechanisms driving symptom overlap between diverse disorders and promises to shine light on common pathophysiological and neurocognitive mechanisms moderating evolving profiles of phenotypic heterogeneity in neurodegenerative conditions.

Our findings hold a number of clinical implications. Transdiagnostic approaches offer a refined accompaniment to categorical diagnostic systems by revealing shared symptomatology across distinct clinical entities. This approach can help refine current diagnostic criteria to accommodate graded performance differences between clinical presentations. Identifying common cognitive and neural disruptions may further aid detection of symptomatic treatment targets applicable to multiple clinical categories. Recent work in PPA, for example, suggests that a combination of speech and language training and excitatory neurostimulation of the parietal cortex may improve naming and verbal fluency performance with sustained benefits for up to 2 weeks [135, 136]. By revealing the neural correlates of general cognitive and language changes, our findings can inform medical and functional restoration programmes aiming to target specific brain networks and regions. Identification of such targets opens further possibilities to harness moderators of phenotype, shared across the PPA spectrum, possibly opening avenues for newer disease management approaches. One such avenue is behavioural treatment, specifically speech and language therapy, which is important to improving overall speech, language abilities, and cognitive communication, in turn benefitting various behavioural domains impacted across the PPA syndromes [137, 138]. These remain important areas of future work.

### Supplementary Information

**Additional file 1: Supplementary Figure 1.** Root-mean-squared-error (RMSE) values derived from four-fold cross-validated (CV) component selection algorithm with venetian blind sampling on behavioural data. Findings suggest that a 4-component solution holds, on average, the lowest RMSE values across 10 iterations of the algorithm. Values >2.975 are truncated for plotting. **Supplementary Figure 2.** Eigenvalues for each component derived from varimax PCA solution on behavioural data in PPA patients. Eigenvalues indicated, rounded to two decimal points. PCA=principal component analysis; PPA=primary progressive aphasia. **Supplementary Figure 3.** Component-specific (yellow) and cumulative variance (blue) explained by the varimax PCA solution on behavioural data in PPA patients. Variance explained amount indicated on top of each bar. PCA=principal component analysis; PPA=primary progressive aphasia. **Supplementary Figure 4.** VBM analyses of whole-brain grey matter atrophy. Panels indicate regions of significant grey matter intensity reduction in each PPA group compared to Controls. Coloured voxels indicate regions that emerged significant in the VBM analyses at *p*<.05 corrected for Family-Wise Error with a cluster threshold of 100 contiguous voxels. Age, total intracranial volume and testing site were included as covariates in all analyses. Clusters are overlaid on the MNI standard brain with *x*, *y*, and *z* co-ordinates reported in MNI standard space. For each cluster, corresponding *t*-values and more details can be found in Supplementary Table 2. L=Left; svPPA=semantic variant primary progressive aphasia; nfvPPA=nonfluent variant primary progressive aphasia; lvPPA=logopenic variant primary progressive aphasia. **Supplementary Figure 5.** Network-based statistics of whole-brain streamline connectivity and fractional anisotropy changes in PPA relative to Controls. Spheres represent nodes from the AAL-116 atlas. Connections represent *t*-values (all surviving *p*_*fwe*_<.05) with thicker lines indicating larger *t*-values. For Streamline Connectivity, all *t*-values exceed 1691.6 for svPPA vs. Controls, 1785.8 for nfvPPA vs. Controls, and 1951.5 for lvPPA vs. Controls. For Fractional Anisotropy, all *t*-values exceed 4311.1 for svPPA vs. Controls, 3889.2 for nfvPPA vs. Controls, and 3302.9 for lvPPA vs. Controls. Age and testing site were included as covariates in all analyses. L=Left; lvPPA=logopenic variant primary progressive aphasia; nfvPPA=nonfluent variant primary progressive aphasia; svPPA=semantic variant primary progressive aphasia. **Supplementary Figure 6.** Network-based statistics of whole-brain streamline connectivity and fractional anisotropy changes between PPA patients. Spheres represent nodes from the AAL-116 atlas. Connections represent *t*-values (all surviving *p*_*fwe*_<.05) with thicker lines indicating larger *t*-values. For Streamline Connectivity, all *t*-values exceed 2934.9 for svPPA<nfvPPA, 2488.5 for svPPA<lvPPA, 3348.6 for lvPPA<nfvPPA, and 3678.4 for nfvPPA<lvPPA. For Fractional Anisotropy, all *t*-values exceed 3905.3 for svPPA<nfvPPA and 3469.8 for nfvPPA<lvPPA. Age and testing site were included as covariates in all analyses. L=Left; svPPA=semantic variant primary progressive aphasia; nfvPPA=nonfluent variant primary progressive aphasia; lvPPA=logopenic variant primary progressive aphasia. **Supplementary Table 1.** Pearson’s correlations between disease duration (symptom duration) and behavioural PCA components. **Supplementary Table 2.** VBM results showing regions of significant grey matter intensity reductions in patient groups versus Controls and between patient group comparisons. **Supplementary Table 3.** VBM results showing regions where grey matter intensity significantly correlates with PCA-generated component performance in the PPA group.

## Data Availability

Ethical requirements to ensure patient confidentiality precludes public archiving of our data but non-identifiable derived data can be provided on request to bona fide researchers. A data transfer agreement may be required if potentially identifiable data are requested, including raw clinical and structural imaging data. Researchers who would like to access data should contact the senior author (M.A.LR).

## Abbreviations

ACE-III: Addenbrooke’s Cognitive Examination
DARTEL: Diffeomorphic anatomical registration through exponentiated lie algebra
FA: Fractional anisotropy
FWE: Family-wise error
MLSE: Mini Linguistic State Examination
MNI: Montreal Neurological Institute
NBS: Network-based statistics
PCA: Principal component analysis
PPA: Primary progressive aphasia
SC: Streamline connectivity
SIFT: Spherical-deconvolution informed filtering of tractograms
TFCE: Threshold-free cluster enhancement
VBM: Voxel-based morphometry
