# Supplementary material for: The neural substrates of transdiagnostic cognitive-linguistic heterogeneity in primary progressive aphasia
Source: Alzheimers Res Ther. 2023 Dec 16;15:219. doi: 10.1186/s13195-023-01350-2 (PMC10724982; doi:10.1186/s13195-023-01350-2)
Supplement: Supplementary file 1 — Additional file 1: Supplementary Figure 1. Root-mean-squared-error (RMSE) values derived from four-fold cross-validated (CV) component selection algorithm with venetian blind sampling on behavioural data. Findings suggest that a 4-component solution holds, on average, the lowest RMSE values across 10 iterations of the algorithm. Values >2.975 are truncated for plotting. Supplementary Figure 2. Eigenvalues for each component derived from varimax PCA solution on behavioural data in PPA patients. Eigenvalues indicated, rounded to two decimal points. PCA=principal component analysis; PPA=primary progressive aphasia. Supplementary Figure 3. Component-specific (yellow) and cumulative variance (blue) explained by the varimax PCA solution on behavioural data in PPA patients. Variance explained amount indicated on top of each bar. PCA=principal component analysis; PPA=primary progressive aphasia. Supplementary Figure 4. VBM analyses of whole-brain grey matter atrophy. Panels indicate regions of significant grey matter intensity reduction in each PPA group compared to Controls. Coloured voxels indicate regions that emerged significant in the VBM analyses at p<.05 corrected for Family-Wise Error with a cluster threshold of 100 contiguous voxels. Age, total intracranial volume and testing site were included as covariates in all analyses. Clusters are overlaid on the MNI standard brain with x, y, and z co-ordinates reported in MNI standard space. For each cluster, corresponding t-values and more details can be found in Supplementary Table 2. L=Left; svPPA=semantic variant primary progressive aphasia; nfvPPA=nonfluent variant primary progressive aphasia; lvPPA=logopenic variant primary progressive aphasia. Supplementary Figure 5. Network-based statistics of whole-brain streamline connectivity and fractional anisotropy changes in PPA relative to Controls. Spheres represent nodes from the AAL-116 atlas. Connections represent t-values (all surviving pfwe<.05) with thicker lines indi [file 13195_2023_1350_MOESM1_ESM.docx]

**SUPPLEMENTARY MATERIAL**

**The neural substrates of transdiagnostic cognitive-linguistic heterogeneity in primary progressive aphasia**

Siddharth Ramanan^1^*, Ajay D. Halai^1^, Lorna Garcia-Penton^1^, Alistair G. Perry^2^, Nikil Patel^3^, Katie A. Peterson^2^, Ruth U. Ingram^4^, Ian Storey^3^, Stefano F. Cappa^5,6^, Eleonora Catricala^5,6^, Karalyn Patterson^1^, James B. Rowe^1,2^, Peter Garrard^3^, and Matthew A. Lambon Ralph^1^

^1^Medical Research Council Cognition and Brain Sciences Unit, University of Cambridge, Cambridge, UK

^2^Department of Clinical Neurosciences and Cambridge University Hospitals NHS Trust, University of Cambridge, Cambridge, UK

^3^Molecular and Clinical Sciences Research Institute, St. George’s, University of London, London, UK

^4^Division of Psychology and Mental Health, University of Manchester, Manchester, UK

^5^IUSS Cognitive Neuroscience Center (ICoN), University Institute of Advanced Studies IUSS, Pavia, Italy

^6^Dementia Research Center, IRCCS Mondino Foundation, Pavia, Italy

**Corresponding author**: Dr. Siddharth Ramanan, MRC Cognition and Brain Sciences Unit, The University of Cambridge, 15 Chaucer Road, Cambridge, CB2 7EF. Email: [Siddharth.ramanan@mrc-cbu.cam.ac.uk](mailto:Siddharth.ramanan@mrc-cbu.cam.ac.uk). Tel: +441223769426

**SUPPLEMENTARY METHODS**

**Image acquisition**

Structural MRI: All participants underwent structural and diffusion MRI of the brain using the following parameters:

- Cambridge: 42 participants (5 svPPA, 9 nfvPPA, 5 lvPPA, 23 Controls) were scanned using a 3T Siemens Skyra MRI scanner. Whole-brain T_1_-weighted structural images were acquired using the following parameters: iPAT2; 208 contiguous sagittal slices; field of view (FOV) = 282 x 282 mm^2^; matrix size 256 x 256; voxel resolution = 1.1 mm^3^; TR/TE/ TI = 2000 ms/2.93 ms/850 ms, respectively; and flip angle 8°. Whole-brain diffusion weighted images were acquired using the following parameters: 64 non-collinear gradient directions, matrix size 96 x 96, 59 slices, TR/TE = 7300/0.09 msec; voxel resolution = 2.5mm^3^, flip-angle = 90°, b-value = 1,000 sec/mm^2^ and five b-value = 0 evenly interspersed across the run, FOV = 240 x 240 mm^2^, in-plane acceleration = 2. Two of these participants (1 lvPPA, 1 nfvPPA) underwent only one set of echo planar imaging, while the rest underwent two sets.
- Manchester: 5 participants (1 svPPA, 2 nfvPPA, 2 lvPPA) were scanned using a 3T Philips Achieva MRI scanner. Whole-brain T_1_-weighted images were acquired using the following parameters: SENSE = 208 contiguous sagittal slices; FOV = 282 x 282 mm^2^; matrix size 256 x 256; voxel resolution = 1.1mm^3^; TR = 6600 ms, TE = 2.99 ms, IT = 850 ms, and flip angle 8°. Whole-brain diffusion weighted images were acquired using the following parameters: 64 non-collinear gradient directions, matrix 94 x 94 mm, 59 slices, TR/TE= 7.3/0.09 msec; slice thickness = 2.5mm, b-value = 1,000 sec/mm2, FOV = 240 x 240 mm^2^.
- St. George’s, University of London: 43 participants (7 svPPA, 4 nfvPPA, 12 lvPPA, 20 Controls) were scanned using a 3T Philips Achieva MRI scanner. Whole-brain T_1_-weighted images were acquired using the following parameters: SENSE = 208 contiguous sagittal slices; FOV = 282 x 282 mm^2^; matrix size 256 x 256; voxel resolution = 1.1mm^3^; TR = 6600-6900 ms, TE = 3.07-3.2 ms, IT = 850 ms, and flip angle 8°. Whole-brain diffusion weighted images were acquired using the following parameters: 64 non-collinear gradient directions, matrix 96 x 96 mm, 59 slices, TR/TE = 7.3/0.09 msec; slice thickness = 2.5mm, b-value = 1,000 sec/mm2, FOV = 240 x 240 mm^2^.

**Behavioural data**

***Data visualisation***

Behavioural findings of group differences and PCA scores were visualised using a combination of box-and-whisker and scatterplots, and colour-blind friendly palettes, all of which are implemented in the *ggplot2* package in R (Wickham, 2016).

***Missing data imputation***

Missing data were imputed using a probabilistic PCA approach. This approach allows for the estimation of the number of underlying principal components in the entire dataset (including missing data) and uses this information to probabilistically predict missing values. As compared to list-wise exclusion of rows of missing data or imputation with central tendency values, probabilistic PCA offers improved stability and guards against overfitting of imputed data points (see Ilin & Raiko, 2010; Tipping & Bishop, 1999). We used the ppca function from MATLAB, where ppca is performed using an isotropic error model, with estimates of missing values determined using an iterative, expectation-maximisation step. The algorithm handles missing values by treating them as additional latent variables. As a result, the reconstructed file (containing estimated values of missing data) corresponds to a low dimensional approximation of the input data (i.e., similar to PCA output) and is equal to the estimated mean (of each variable in the input data) + principal component scores * principal component coefficients (from the probabilistic PCA analysis). More details on this function are available in the MATLAB description of ppca: <https://ww2.mathworks.cn/help/stats/ppca.html>.

For the probabilistic PCA, we first standardized all available data (*z*-scored to group mean). Then, we used a *k*-fold cross-validation approach (with *k* = 4) where the dataset was first divided into three ‘training’ sets and one ‘testing’ set (as *k* = 4). Following this, a *k*-fold cross-validation probabilistic PCA with 1,000 permutations was used to estimate the component solution (*i.e.,* the number of components) that represent the underlying structure of the data. The solution with the lowest root-mean-square-error for all held-out cases (i.e., testing set cases) over 1,000 permutations was considered the most ‘stable’ solution (Ballabio, 2015). This approach was also used to select the optimal number of components for subsequent PCA on the imputed dataset. All imputed scores were visually inspected to ensure they fit with each patient’s overall cognitive profile.

***Component selection for behaviour data***

In addition to relying on recommended criteria (e.g., eigenvalues ≥~1.0) (Jolliffe, 2005), we conducted data-driven component selection to determine the optimal number of components to be extracted using PCA. Using a freely available MATLAB software (Ballabio, 2015), component selection was undertaken using four-fold cross-validation (10 iterations) with venetian-blind sample shuffling at each iteration to control for participant order effects. In each iteration: (i) data were split into three-train and one-test fold; (ii) PCA models were built on train folds, removing one variable at a time, and (iii) model values were inputted into linear regressions to predict scores in left-out test fold, with prediction accuracy for each model estimated by root-mean-squared-error (RMSE) values. The solution with the lowest average RMSE values across iterations emerged as the winning model for number of components underlying the dataset.

**Connectome data**

***Structural connectome construction for streamline count (SC) and fractional anisotropy (FA)***

To calculate streamline count for each individual, we used the *tck2connectome* command, superimposing their individualised AAL brain parcellation on their whole brain tractogram (post SIFT2) file and calculated a connectivity matrix of streamline counts between AAL-116 nodal pairs. For FA, for each streamline, the value of the underlying scalar FA image was sampled at each vertex, following which a single mean value per streamline was generated using the *tcksample* command. Then, the product of the magnitude of contribution of each streamline to the matrix and the mean FA index value for that streamline was derived. Finally, the *tck2connectome* command was used to calculate mean scalar values for each connectome edge across the values of the “mean FA” contributed by all streamlines assigned to that particular edge (<https://mrtrix.readthedocs.io/en/latest/reference/commands/tck2connectome.html>). The final connectomes were unthresholded to the strongest weights. For each index (SC, FA), the output for each connectome was a 116*116 matrix of values.

**Supplementary Results**

**Group differences on MLSE performance**

Significant group differences were noted on the MLSE total score, with all patient groups performing significantly poorer than Controls (all *p*<.001). On this total score, patient groups performed comparably to each other (all *p*>.1). Examining MLSE subdomains, significant group differences were noted on MLSE Motor Speech total, where lvPPA and nfvPPA groups performed significantly worse than Controls (both *p*<.001). Direct comparisons between patient groups revealed disproportionately greater Motor Speech deficits in nfvPPA, relative to lvPPA and svPPA groups (both *p*<.001). On MLSE Phonological Structure total, significant group differences were noted, with all patient groups performing significantly poorer than Controls (all *p*<.005), but comparably to each other (all *p*>.1). Significant group differences were also noted on MLSE Semantic Knowledge total, with all patient groups again performing poorer than the Control group (all *p*<.001). On this domain, comparisons between patient groups revealed the svPPA group to show disproportionately poorer performance when compared to lvPPA and nfvPPA (both *p*<.025). Examining MLSE Syntactic Knowledge total, PPA groups displayed significantly greater deficits relative to Controls (all *p*<.001), however, no post-hoc comparisons revealed no significant differences between patient groups (all *p*>.07). Finally, on the MLSE Auditory-Verbal Working Memory total, significant group differences were noted, lvPPA and svPPA patients performing significantly worse than Controls (both *p*<.001). Direct contrasts between PPA groups revealed significantly greater auditory-verbal working memory impairments in the lvPPA group relative to the nfvPPA group (*p*<.001).

**Group differences in white matter integrity (NBS)**

Considering SC first, relative to Controls, svPPA displayed significantly reduced connectivity to a network of 202 connections (all *p*_fwe_<.05). Structural disconnections were maximal at the anterior temporal lobe with further reductions in connectivity between temporal lobes and with frontoparietal (Supplementary Figure 5). In comparison to Controls, the nfVPPA group displayed significantly reduced connectivity to a network of 273 connections (all *p*_fwe_<.05) centered around frontoinsular, superior/medial frontal, and dorsolateral frontal cortices, and their connections with middle temporal and parieto-occipital cortices (Supplementary Figure 5). Relative to Controls, the lvPPA group displayed significantly reduced connectivity in a network of 565 connections (all *p*_fwe_<.05). Of the patient-Control comparisons, lvPPA showed the most widespread reductions in SC with maximal effects noted at the left temporoparietal/inferior parietal cortex and its connectivity with temporal, superior frontal, parieto-occipital, and cerebellar nodes (Supplementary Figure 5). Between patient comparisons revealed svPPA patients to show significantly fewer streamline connections in the left anterior temporal lobes when compared to nfvPPA (17 connections) and lvPPA (6 connections) (Supplementary Figure 6). Relative to nfvPPA, lvPPA patients displayed significantly reduced connectivity to a network of 7 connections from parietal to ventral frontal and parietal to cerebellar regions. The reverse contrast revealed significantly reduced connectivity between left lateral prefrontal and insular cortices in nfvPPA, relative to lvPPA (all *p*_fwe_<.05).

Turning to FA, findings from each group concurred with their respective SC results but further extended to include a wider network of disconnections between bilateral frontal, temporal, parietal, occipital, and cerebellar regions. Relative to Controls, svPPA displayed significantly decreased FA to 1,163 connections (all *p*_fwe_<.05) with maximal effects at anterior/ventral temporal cortices. In comparison to Controls, nfvPPA patients showcased reduced FA in a network of 1,984 connections (all *p*_fwe_<.05) with maximal changes noted at left fronto-insular and dorsolateral frontal regions. Finally, lvPPA patients displayed significant alterations to structural connectivity to a network of 1189 connections as opposed to Controls, with greatest effects noted at the left temporoparietal junction/inferior parietal cortex, and its connections to right parietal, bilateral prefrontal and temporal, and cerebellar regions. Direct comparisons between patient groups revealed svPPA patients, when compared to nfvPPA, showed significantly reduced FA between right anterior temporal and frontoinsular regions (10 connections) (Supplementary Figure 6). Compared to lvPPA, nfvPPA showed significantly reduced FA between cortical midline and bilateral lateral frontal regions, and between left insular and right ventral temporal regions (6 connections, Supplementary Figure 6). No significant results emerged for any other contrasts.


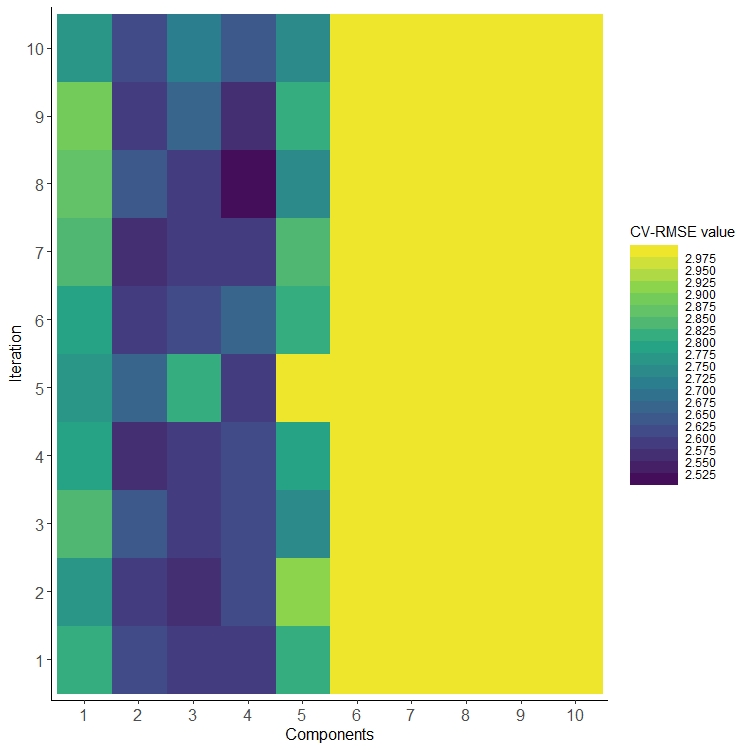


**Supplementary Figure 1**. Root-mean-squared-error (RMSE) values derived from four-fold cross-validated (CV) component selection algorithm with venetian blind sampling on behavioural data. Findings suggest that a 4-component solution holds, on average, the lowest RMSE values across 10 iterations of the algorithm. Values >2.975 are truncated for plotting.


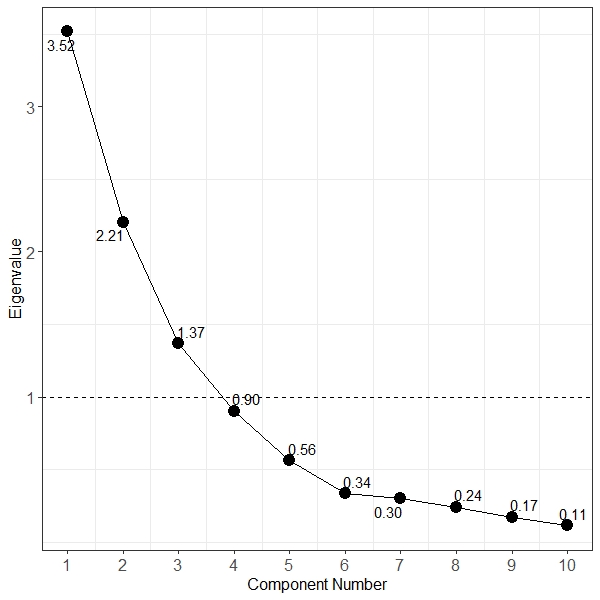


**Supplementary Figure 2**. Eigenvalues for each component derived from varimax PCA solution on behavioural data in PPA patients. Eigenvalues indicated, rounded to two decimal points. PCA=principal component analysis; PPA=primary progressive aphasia.

**
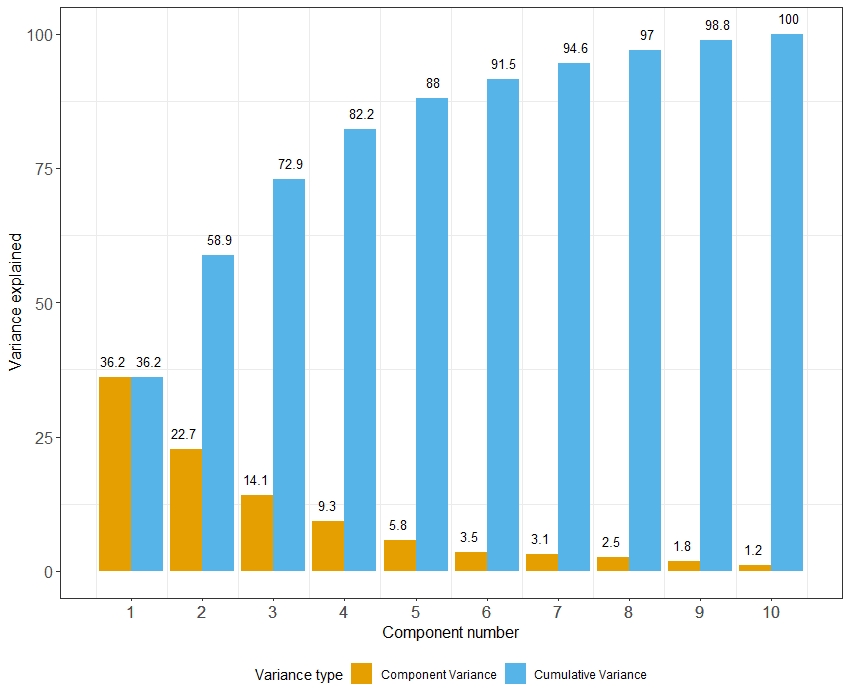
**

**Supplementary Figure 3.** Component-specific (yellow) and cumulative variance (blue) explained by the varimax PCA solution on behavioural data in PPA patients. Variance explained amount indicated on top of each bar. PCA=principal component analysis; PPA=primary progressive aphasia.


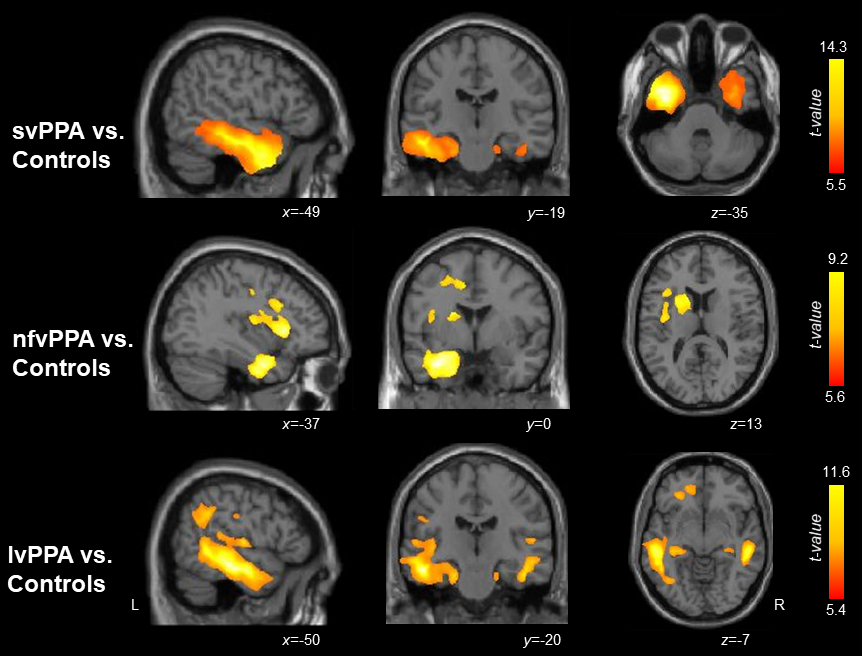


**Supplementary Figure 4. VBM analyses of whole-brain grey matter atrophy.** Panels indicate regions of significant grey matter intensity reduction in each PPA group compared to Controls. Coloured voxels indicate regions that emerged significant in the VBM analyses at *p*<.05 corrected for Family-Wise Error with a cluster threshold of 100 contiguous voxels. Age, total intracranial volume and testing site were included as covariates in all analyses. Clusters are overlaid on the MNI standard brain with *x*, *y*, and *z* co-ordinates reported in MNI standard space. For each cluster, corresponding *t*-values and more details can be found in Supplementary Table 2. L=Left; svPPA=semantic variant primary progressive aphasia; nfvPPA=nonfluent variant primary progressive aphasia; lvPPA=logopenic variant primary progressive aphasia.


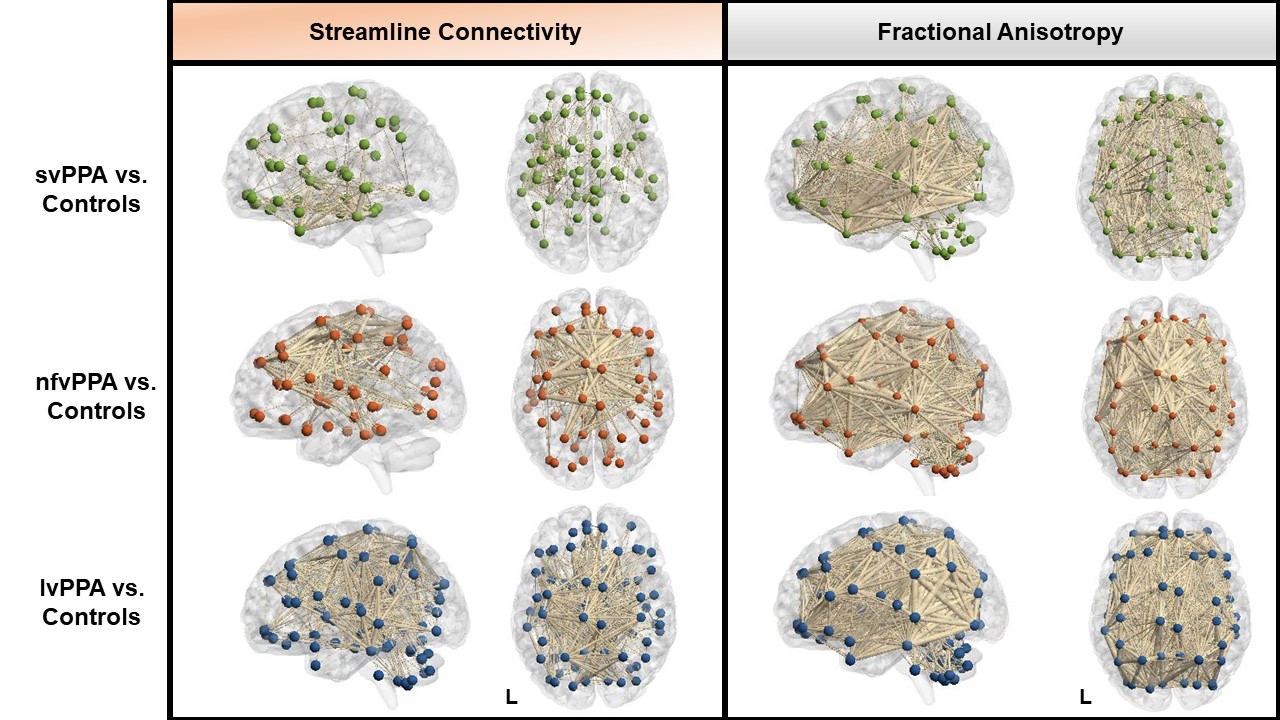


**Supplementary Figure 5**. **Network-based statistics of whole-brain streamline connectivity and fractional anisotropy changes in PPA relative to Controls.** Spheres represent nodes from the AAL-116 atlas. Connections represent *t*-values (all surviving *p_fwe_*<.05) with thicker lines indicating larger *t*-values. For Streamline Connectivity, all *t*-values exceed 1691.6 for svPPA vs. Controls, 1785.8 for nfvPPA vs. Controls, and 1951.5 for lvPPA vs. Controls. For Fractional Anisotropy, all *t*-values exceed 4311.1 for svPPA vs. Controls, 3889.2 for nfvPPA vs. Controls, and 3302.9 for lvPPA vs. Controls. Age and testing site were included as covariates in all analyses. L=Left; lvPPA=logopenic variant primary progressive aphasia; nfvPPA=nonfluent variant primary progressive aphasia; svPPA=semantic variant primary progressive aphasia.


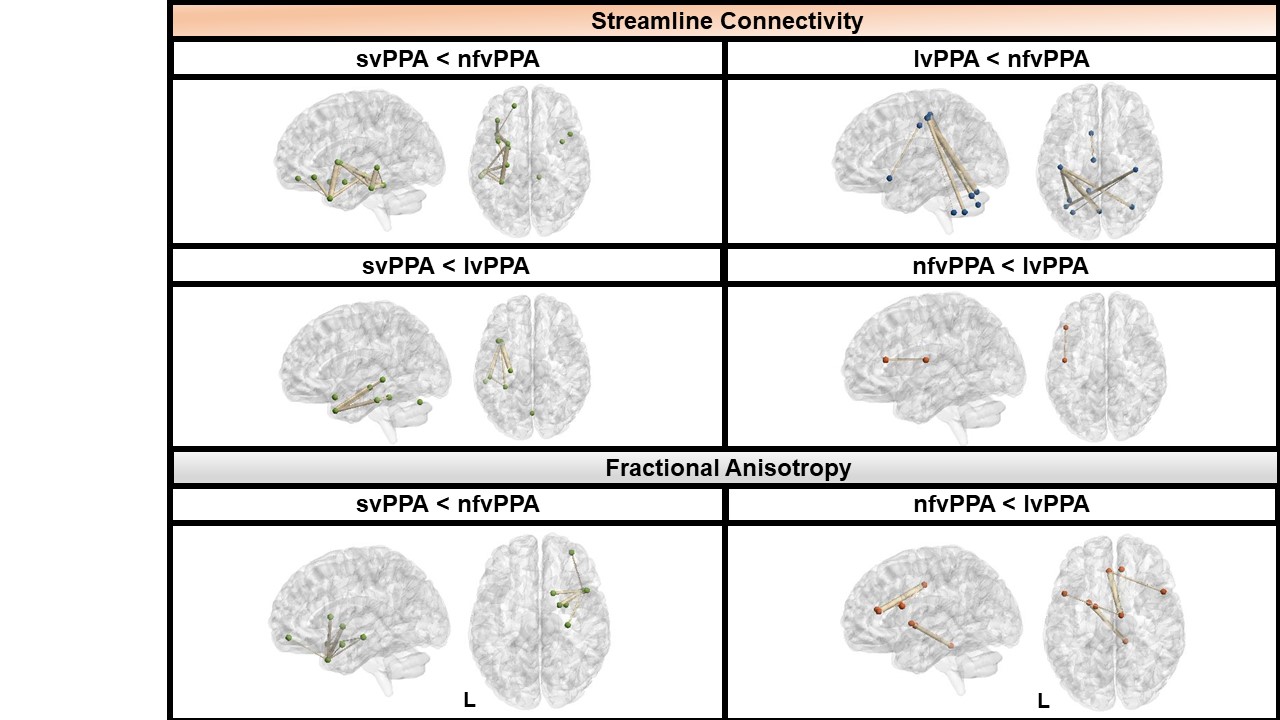


**Supplementary Figure 6.** **Network-based statistics of whole-brain streamline connectivity and fractional anisotropy changes between PPA patients.** Spheres represent nodes from the AAL-116 atlas. Connections represent *t*-values (all surviving *p_fwe_*<.05) with thicker lines indicating larger *t*-values. For Streamline Connectivity, all *t*-values exceed 2934.9 for svPPA<nfvPPA, 2488.5 for svPPA<lvPPA, 3348.6 for lvPPA<nfvPPA, and 3678.4 for nfvPPA<lvPPA. For Fractional Anisotropy, all *t*-values exceed 3905.3 for svPPA<nfvPPA and 3469.8 for nfvPPA<lvPPA. Age and testing site were included as covariates in all analyses. L=Left; svPPA=semantic variant primary progressive aphasia; nfvPPA=nonfluent variant primary progressive aphasia; lvPPA=logopenic variant primary progressive aphasia.

**Supplementary Table 1. Pearson’s correlations between disease duration (symptom duration) and behavioural PCA components**

| **Component** | **Correlation value** |
| --- | --- |
| C1: General Cognition | *r*=.009; *p*=.95 |
| C2: Semantic memory | *r*=-.24; *p*=.1 |
| C3: Working memory | *r*=.05; *p*=.7 |
| C4: Motor Speech/Phonology. | *r*=.11; *p*=.43 |

*Note. r* values indicate correlation strength as derived from two-tailed Pearson’s correlations.

**Supplementary Table 2. VBM results showing regions of significant grey matter intensity reductions in patient groups versus Controls and between patient group comparisons.**

| Regions | Side | Number of voxels | Peak MNI co-ordinates | | | *t*-value |
| --- | --- | --- | --- | --- | --- | --- |
|  |  |  | *x* | *y* | *z* |  |
| *svPPA vs. Controls* | | | | | | |
| Temporal pole extending ventrally into fusiform and parahippocampal gyrus, hippocampus and amygdala, inferior/middle/superior temporal gyrus, inferior/middle occipital gyrus, cerebellum | Left | 63,322 | -37 | -5 | -35 | 14.3 |
| Temporal pole, extending laterally into inferior/middle/superior temporal gyrus, and ventrally into fusiform gyrus, parahippocampal gyrus, hippocampus and amygdala, and cerebellum | Right | 16,103 | 36 | 6 | -27 | 7.7 |
| Caudate | Left | 556 | -15 | -7 | 22 | 5.9 |
| Middle/inferior temporal gyrus and fusiform gyrus | Right | 124 | 50 | -39 | -14 | 5.5 |
|  |  |  |  |  |  |  |
| *nfvPPA vs. Controls* | | | | | | |
| Parahippocampal gyrus, hippocampus extending into inferior/middle/superior temporal gyrus and temporal pole | Left | 13,936 | -34 | 0 | -28 | 9.2 |
| Precentral gyrus, middle/superior frontal gyrus, anterior cingulate cortex | Left | 5,088 | -15 | 8 | 43 | 6.9 |
| Inferior/middle frontal gyrus and insula | Left | 4,868 | -37 | 21 | 4 | 8.2 |
| Caudate and putamen | Left | 2,895 | -25 | 9 | 14 | 6.8 |
| Inferior/middle frontal gyrus and insula | Right | 275 | 37 | 14 | 7 | 5.6 |
| Supramarginal gyrus | Left | 132 | -45 | -35 | 33 | 5.6 |
|  |  |  |  |  |  |  |
| *lvPPA vs. Controls* | | | | | | |
| Temporoparietal junction, superior/middle/inferior temporal gyrus and inferior parietal lobe (supramarginal and angular gyrus) extending anterio-ventrally into parahippocampal gyrus, caudate, fusiform gyrus, temporal pole and extending caudally into middle occipital gyrus and cerebellum | Left | 55,170 | -49 | -20 | -19 | 11.6 |
| Superior/middle/inferior temporal gyrus, inferior parietal lobe (supramarginal and angular gyrus), parahippocampal gyrus, hippocampus, amygdala extending dorsally into insula and caudally into occipital cortex and cerebellum | Right | 10,797 | 51 | -33 | -12 | 10 |
| Supramarginal and angular gyrus | Left | 5,483 | -46 | -48 | 26 | 8.6 |
| Caudate, putamen and thalamus | Left | 4,496 | -14 | 1 | 20 | 7.4 |
| Superior/middle/inferior frontal gyrus, anterior cingulate cortex, medial frontal gyrus, orbitofrontal cortex | Left | 3,427 | -25 | 34 | -12 | 6.9 |
| Caudate and putamen | Right | 2,247 | 15 | 7 | 18 | 6.6 |
| Insula, precentral and postcentral gyrus | Left | 1,824 | -41 | -10 | 20 | 6.7 |
| Amygdala, hippocampus and parahippocampal gyrus | Right | 1,795 | 25 | -5 | -21 | 6.2 |
| Hippocampus, caudate and thalamus | Right | 1,410 | 29 | -35 | 7 | 6.8 |
| Superior temporal gyrus | Right | 519 | 52 | -21 | 9 | 6.3 |
| Middle/inferior temporal gyrus | Right | 328 | 46 | -56 | -3 | 5.8 |
| Angular and supramarginal gyrus | Left | 278 | 45 | -37 | 30 | 5.4 |
|  |  |  |  |  |  |  |
| *svPPA vs. nfvPPA* | | | | | | |
| No significant clusters |  |  |  |  |  |  |
|  |  |  |  |  |  |  |
| *nfvPPA vs. svPPA* | | | | | | |
| No significant clusters |  |  |  |  |  |  |
|  |  |  |  |  |  |  |
| *svPPA vs. lvPPA* | | | | | | |
| No significant clusters |  |  |  |  |  |  |
|  |  |  |  |  |  |  |
| *lvPPA vs. svPPA* | | | | | | |
| No significant clusters |  |  |  |  |  |  |
|  |  |  |  |  |  |  |
| *lvPPA vs. nfvPPA* | | | | | | |
| No significant clusters |  |  |  |  |  |  |
|  |  |  |  |  |  |  |
| *nfvPPA vs. lvPPA* | | | | | | |
| No significant clusters |  |  |  |  |  |  |

*Note*. Clusters presented above emerged significant in the VBM analyses at a threshold of *p*<.05 corrected for Family-Wise Error rate with a cluster threshold of 100 contiguous voxels. Age, total intracranial volume and testing site were included as covariates in the analyses. Visual depiction of clusters can be found in Supplementary Figure 4. svPPA=semantic variant primary progressive aphasia; nfvPPA=nonfluent variant primary progressive aphasia; lvPPA=logopenic variant primary progressive aphasia.

**Supplementary Table 3. VBM results showing regions where grey matter intensity significantly correlates with PCA-generated component performance in the PPA group.**

| Regions | Side | Number of voxels | Peak MNI co-ordinates | | | *t*-value |
| --- | --- | --- | --- | --- | --- | --- |
|  |  |  | *x* | *y* | *z* |  |
| *General cognition component (Component 1)* | | | | | | |
| Precuneus, left angular gyrus, left supramarginal gyrus, posterior cingulate, superior parietal cortex | Bilateral | 10,966 | -12 | -63 | 39 | 5.2 |
| Angular gyrus, supramarginal gyrus, superior/middle temporal gyrus | Left | 6,111 | -46 | -57 | 32 | 4.8 |
| Inferior/middle occipital and temporal gyrus | Right | 3,451 | 26 | -82 | -4 | 5.3 |
| Caudate, putamen and globus pallidus |  | 3,360 | 18 | -5 | 22 | 4.7 |
| Middle occipital and lingual gyrus | Left | 1,812 | -17 | -80 | -5 | 4.4 |
| Supramarginal gyrus, postcentral gyrus | Right | 1,587 | 39 | -34 | 40 | 4 |
| Middle occipital and temporal gyrus | Left | 1,482 | -39 | -67 | -5 | 5.1 |
| Postcentral gyrus, superior parietal cortex | Right | 510 | 27 | -41 | 58 | 4 |
| Superior occipital gyrus | Right | 393 | 15 | -82 | 12 | 3.8 |
| Thalamus | Right | 212 | 9 | -7 | 12 | 3.5 |
| Supramarginal gyrus | Right | 156 | 44 | -30 | 21 | 3.4 |
| Middle frontal gyrus | Right | 120 | 50 | 38 | 20 | 3.6 |
| Superior temporal gyrus | Left | 101 | -56 | -20 | 9 | 3.7 |
| Inferior frontal gyrus | Left | 96 | -43 | 6 | 23 | 3.6 |
|  |  |  |  |  |  |  |
| *Semantic memory component (Component 2)* | | | | | | |
| Anterior temporal lobe, inferior/middle/superior temporal gyrus extending ventrally into fusiform gyrus, parahippocampal gyrus, hippocampus, amygdala and caudally into cerebellum | Left | 59,948 | -51 | -4 | -26 | 7.2 |
| Anterior temporal lobe, inferior/middle/superior temporal gyrus extending ventrally into fusiform gyrus, parahippocampal gyrus, hippocampus, amygdala and caudally into brain stem | Right | 28,034 | 20 | 4 | -49 | 5.2 |
| Thalamus, caudate, globus pallidus and hypothalamus | Left | 2,062 | -8 | 3 | 4 | 4.1 |
| Medial/inferior frontal gyrus and anterior cingulate cortex | Left | 1,018 | -12 | 22 | -17 | 3.8 |
| Medial/inferior frontal gyrus and orbitofrontal cortex | Right | 227 | 13 | 25 | -17 | 3.5 |
| Medial/inferior frontal gyrus, anterior cingulate cortex and orbitofrontal cortex | Left | 149 | -8 | 34 | -9 | 3.4 |
|  |  |  |  |  |  |  |
| *Working memory component (Component 3)* | | | | | | |
| Supramarginal gyrus, angular gyrus and superior temporal gyrus | Left | 329 | -53 | -55 | 38 | 3.6 |
| Superior/middle temporal gyrus | Left | 187 | -54 | -49 | 8 | 3.7 |
| Angular gyrus | Left | 68 | -26 | -64 | 17 | 3.8 |
|  |  |  |  |  |  |  |
| *Motor Speech/Phonology component (Component 4)* | | | | | | |
| Middle/inferior frontal gyrus, precentral gyrus | Right | 567 | 35 | 3 | 41 | 3.8 |
| Superior frontal gyrus | Left | 178 | -2 | 5 | 59 | 3.8 |
| Inferior frontal gyrus and precentral gyrus | Left | 78 | -48 | 12 | 16 | 3.4 |

*Note*. Clusters presented above emerged significant in the VBM analyses at a threshold of *p*<.001 uncorrected with a cluster threshold of 50 contiguous voxels. Age, total intracranial volume and testing site were included as covariates in the analyses. Visual depiction of clusters can be found in main manuscript, Figures 3-5. VBM=voxel-based morphometry; PCA= principal component analysis; PPA=primary progressive aphasia.

**Supplementary references**

Ballabio, D. (2015). A MATLAB toolbox for Principal Component Analysis and unsupervised exploration of data structure. *Chemometrics and Intelligent Laboratory Systems, 149*, 1-9. doi:10.1016/j.chemolab.2015.10.003

Ilin, A., & Raiko, T. (2010). Practical Approaches to Principal Component Analysis in the Presence of Missing Values. *Journal of Machine Learning Research, 11*, 1957-2000.

Jolliffe, I. (2005). Principal component analysis. *Encyclopedia of statistics in behavioral science*.

Tipping, M. E., & Bishop, C. M. (1999). Probabilistic principal component analysis. *Journal of the Royal Statistical Society Series B-Statistical Methodology, 61*, 611-622. doi:Doi 10.1111/1467-9868.00196

Wickham, H. (2016). ggplot2: Elegant Graphics for Data Analysis. New York: Springer-Verlag. Retrieved from <https://ggplot2.tidyverse.org>
